# Genetic Background Drives Transcriptional Variation in Human Induced Pluripotent Stem Cells

**DOI:** 10.1371/journal.pgen.1004432

**Published:** 2014-06-05

**Authors:** Foad Rouhani, Natsuhiko Kumasaka, Miguel Cardoso de Brito, Allan Bradley, Ludovic Vallier, Daniel Gaffney

**Affiliations:** 1 Wellcome Trust Sanger Institute, Hinxton, Cambridge, United Kingdom; 2 University of Cambridge, Cambridge, Cambridge, United Kingdom; Georgia Institute of Technology, United States of America

## Abstract

Human iPS cells have been generated using a diverse range of tissues from a variety of donors using different reprogramming vectors. However, these cell lines are heterogeneous, which presents a limitation for their use in disease modeling and personalized medicine. To explore the basis of this heterogeneity we generated 25 iPS cell lines under normalised conditions from the same set of somatic tissues across a number of donors. RNA-seq data sets from each cell line were compared to identify the majority contributors to transcriptional heterogeneity. We found that genetic differences between individual donors were the major cause of transcriptional variation between lines. In contrast, residual signatures from the somatic cell of origin, so called epigenetic memory, contributed relatively little to transcriptional variation. Thus, underlying genetic background variation is responsible for most heterogeneity between human iPS cell lines. We conclude that epigenetic effects in hIPSCs are minimal, and that hIPSCs are a stable, robust and powerful platform for large-scale studies of the function of genetic differences between individuals. Our data also suggest that future studies using hIPSCs as a model system should focus most effort on collection of large numbers of donors, rather than generating large numbers of lines from the same donor.

## Author Summary

Human induced pluripotent stem (hiPS) cells are a potentially powerful model system for studying human disease and development, and a resource for personalized medicine. However, it has been reported that hiPS cells exhibit substantial heterogeneity which could limit their use as model systems. Clearly, knowledge of the source of heterogeneity is key for deeper understanding of the use of human iPS cells for basic and therapeutic applications. One source of this heterogeneity has been presumed to be “memory” of the adult somatic cell from which the hIPS cells were derived, but the evidence to support this view is scant. We have generated a set of human iPS cells from a set of somatic cell types from different donors. Our study shows that cell lines from different somatic sources but from the same donor (i.e. with the same genome) are more similar than cell lines isolated from the same tissue type but from different donors. Once genetic changes are accounted for, all aspects of gene expression, including mRNA levels, splicing and imprinting are highly similar between iPS cells derived from different human tissues. Thus, most of the previously described transcriptional variation between cell lines is likely to be genetic in origin.

## Introduction

Induced pluripotent stem cells (iPSCs) are the subject of tremendous interest as model systems for studying human disease and development [1], [2]. However, cellular reprogramming to iPSCs is an inefficient process in which stochastic events during clonal selection may fix a variety of alternative epigenetic and transcriptional states[3]. Some reports have described significant variation between iPS cells and ES cells, while others have suggested that iPS cells retain a memory of the somatic tissue from which they were derived that may negatively affect their differentiation efficiency into certain cell lineages [4]–[10]. However, comparisons between human iPS cells and ES cells are confounded with differences in genetic background because the lines are derived from different donors. Likewise, because collection of multiple primary tissues from the same individual is frequently impractical, studies of cellular memory in hiPS cells have often confounded iPS source tissue type and donor genetic background. This is important because many cellular phenotypes, including transcription and methylation, are substantially impacted by genetic differences between individuals [11]–[13].

In this study we set out to understand the basis of this variation by establishing a set of iPS cells from a panel of tissues isolated in parallel from several different donors. RNA-seq data sets from these lines, the corresponding adult somatic cells and human ES cells have been systematically compared. This has enabled us to investigate patterns of expression, splicing and imprinting between these iPS cells, their adult cell progenitors and compare these with hES cells. Using a statistical model we estimated the relative contributions of genetic background and tissue of origin to transcriptional variability between human iPS cell lines.

## Results

**Figure 1:**
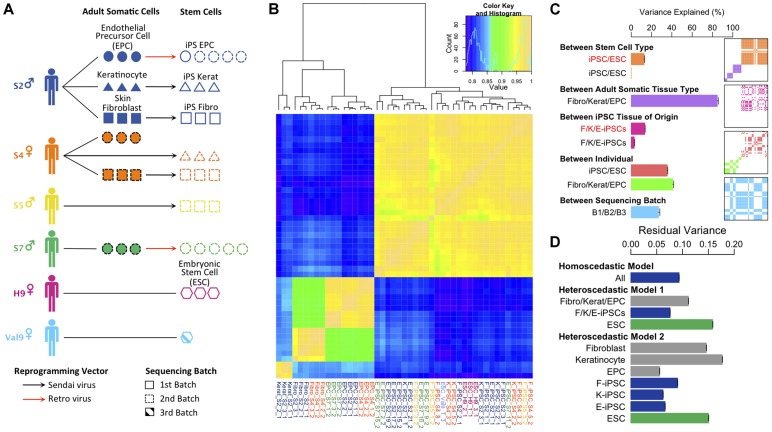
Experimental design and variance components analysis of transcription in iPSCs, somatic progenitors, H9 and Val9 embryonic stem cells. (a) Schematic showing experimental design. (b) Heatmap of Pearson correlation coefficients of log2 FPKMs (fragments per kilobase of exon per million fragments mapped) across all genes in all samples (N = 47). Complete-linkage clustering on the correlation coefficients was performed to order samples by similarity. (c) Decomposition of transcriptional variation using a linear mixed model. Each bar shows the percentage of transcriptional variance explained (%VE) by each of the components, holding all other factors constant (see Text S1 section 1.3 for details). Bars show the percentage variance explained between iPS and ES cells δ^2^_22_/(δ^2^_22_+σ^2^) (orange); between adult tissues δ^2^_21_/(δ^2^_21_+σ^2^) (purple); between iPSC somatic tissue of origin δ^2^_32_/(δ^2^_32_+σ^2^) (pink); between individual (iPSCs) δ^2^_41_/(δ^2^_42_+σ^2^) (red); between individual (adult tissues) δ^2^_41_/(δ^2^_41_+σ^2^) (green) and between sequencing batches δ^2^_5_/(δ^2^_5_+σ^2^)(blue). δ^2^ and σ^2^ parameters are explained in section 1.3 of Text S1. Red text denotes variance explained by a component in a model that excluded an individual component, black text denotes variance explained in a model including an individual component. Panels show layered covariance matrices Z_j_ D_j_ Z_j_ for the variance components j (j = 2…5) of which the correlation heatmap in Figure 1b consists. The color corresponds to each of variance components in the barchart of Figure 1c and white color corresponds to covariance of 0. The sample order of the layered matrices is compatible with Figure 1b. (d) Bars show the estimate of residual variance parameters estimated from a homoscedastic and two heteroscedastic models. “Homoscedastic model” represents a model with a single error term shared over all samples in the data set, and the estimate of this error term is labeled “All”. The heteroscedastic models 1 and 2 are described in the main text and in the Supplement (Section 1.3.3. “Heteroscedastic Model”. Each bar shows the estimate of a residual error parameter as outlined the Supplement section 1.3.3.

We established primary fibroblast, keratinocyte and endothelial progenitor cell (EPC) somatic cell lines from three healthy male organ donors, labeled S2, S5 and S7, and one healthy female donor (S4). From each primary adult tissue cell line, we derived at least three independent iPS cell lines for each donor. For the adult cell cultures we extracted RNA following each of three passages to give a total of 18 RNA samples from adult donor cells (6 fibroblast, 3 keratinocyte and 9 EPCs). We also extracted RNA from the iPS cell lines derived from each of these tissues to give a total of 9 RNA samples from fibroblast-derived iPSCs (F-iPSCs), 6 from keratinocyte-derived iPSCs (K-iPSCs) and 10 EPC-derived iPSCs (E-iPSCs). Finally, we also extracted 4 RNA samples from two ES cell lines, H9 and Val9. Throughout our study highly standardized conditions were used. This included the isolation, derivation and culture of primary cell cells as well as use of the same batches of reprogramming viruses, serum and media. Each of the primary cell lines were reprogrammed using the four Yamanaka factors to generate a series of 25 iPS cell lines derived from each of the three adult tissues (hereafter F-, K- and E-iPS cells). The differentiation properties of our iPS cell lines were checked by differentiating a subset to endoderm, mesoderm and neuroectoderm derived cell types (Figure S1). Polyadenylated mRNA was prepared from each iPS cell line after 10 passages, the somatic cell lines at passage 3 and from two human ES cell lines which was subjected to high-depth RNA-seq (Fig. 1a). We checked a subset of lines for Sendai virus persistence using RT-PCR with virus-specific primers, but found no evidence for viral presence following passage (Figure S2). For each sample, we performed 75 bp paired end sequencing on the Illumina HiSeq2000 platform. In total we generated 7.3 billion reads, with between 85.3 and 229.8 million reads sequenced in each sample (Figure S3). We mapped reads to assembly h37 of the human genome using Bowtie2 [14] and constructed spliced alignments using Tophat2 [15]. Following read alignment and QC filtering, between 49% and 89% of reads mapped uniquely to the human genome (Figure S4).

Initially we examined the global pattern of transcription in the different cell lines. Previous work has suggested that embryonic stem cells are more transcriptionally active than differentiated cells [16]. Within protein coding regions there was a clear bimodal distribution of gene expression levels in all samples reflecting abundantly expressed and transcriptionally repressed genes (Figure S5). Adult cell lines exhibited more completely repressed and very highly expressed genes compared with hiPS cells and hES. Relative to differentiated lineages approximately twice as many genes could be classified as coming from the repressed mode of the distribution in the adult cell lines compared with hiPS cells and ES cells (Figure S 5, inset). We also found that more transcription appeared to be originate from repetitive elements in pluripotent stem cells (Figure S6), although it is unclear whether this arises from a slightly more relaxed chromatin structure in stem cells, or is an artifact resulting from the slight excess of very highly expressed genes in somatic cells relative to pluripotent stem cells.

### Variance component analysis of transcription levels

Next, we sought to quantify the contribution of multiple biological and experimental factors to transcriptional variation in hiPSCs. Hierarchical clustering clearly placed adult somatic cells and pluripotent cells in two distinct clades (Fig. 1b). However other sources of variation, such as tissue of origin, were more difficult to discern. To quantify the relative importance of different sources of global transcriptional variation more precisely we employed a variance component analysis. Here, transcriptional variation was decomposed into five separate components. These components comprised: (1) a random intercept term (2) a component to capture variation in transcription between the three adult somatic tissues, hESCs and hiPSCs (3) a component modeling differences between F-, K- and E-iPSCs (4) a component capturing transcriptional variation between different donors or genetic backgrounds and (5) a component captures differences between the two sequencing batches in our data set (see Text S1). We quantified the contribution of each of the five components using intraclass correlation, which measures the proportion of total transcriptional variance explained (VE) by different experimental groups holding other model factors constant. As such, the estimated VEs for each component are not constrained to sum to 100%. Throughout, we modeled the effect of sequencing batch to disentangle its potential influence from the other variance components in the model.

We found that inter-individual transcriptional variation in hiPS cells (VE ∼38%) is considerably larger than that between somatic tissue of origin (VE∼4%) with an even smaller fraction of transcriptional variation (<1%) explained by differences between iPSCs and ESCs (Fig. 1c). Strikingly, when we didn't correct for variation between individuals, transcriptional variation between iPSCs and ESCs and between different iPSC tissues of origin appeared to be much larger (<1% vs 12.7%, iPSCs vs ESCs, 4% vs 13.5%, iPSC tissue of origin, with versus without individual included in the model, respectively) (Fig1c). This suggests that some previous observations of cellular memory and transcriptional differences between iPSCs and ESCS may in fact arise from changing genetic backgrounds rather than experimental effects. Confounding with donor genetic background seems particularly plausible for comparisons between iPSCs and ESCs where controlling for genetic differences is often impossible [4], [17], or in cases where iPSC tissue of origin is confounded with donor genetic background [9], [18]. We found that fibroblast- and keratinocyte-derived iPS cells (F- and K-iPSCs) were highly similar at the transcriptional level with tissue of origin explaining <1% of the transcriptional variation in the between them. In fact, the majority of the tissue of origin effect we observe arises from differences between F/K-iPS cells and EPC-derived iPS cells (E-iPS cells). Some of this signal could reflect transcriptional memory. However it is possible that the reprogramming method could account for this difference because the EPCs were resistant to Sendai virus infection and were therefore derived using the retroviral method. Individual transcriptional variation was slightly greater in adult somatic cells (VE∼42%) than in stem cells. We speculate that this could potentially be explained by non-genetic differences between individuals, such as varying methylation status, which are present in somatic cells but erased during cellular reprogramming.

We also examined the amount of residual transcriptional variation that remained unexplained by any of the known factors using a heteroscedastic model (Text S1). This extension of the model allowed us to capture differences in residual variance between different subsets of our data set. In our experiment, the residual variance captures transcriptional variation between different cell lines from the same donor, either as growths of different IPS cell lines derived from the same donor, from different growths of either the ESCs or as different passages of adult cells from the same donor. We compared two heteroscedastic models with a homoscedastic model, which contained a single error term for the entire data set (Figure 1d, “All”). Heteroscedastic model 1 (“model 1”) contained three terms representing variation between biological replicates of adult cells, IPS cells and ESCs. The results of this analysis illustrate that the residual variation between replicates of IPS cells is lower than that in adult cells and ESCs (Figure 1d: Heteroscedastic Model 1 “F/K/E-iPSCs” versus “ESC”). Heteroscedastic model 2 further divided error into components for each of the three different adult cell types, for each of the three IPS cell types and for ESCs. This analysis demonstrated that variation between biological replicates of IPS lines derived from different tissues was relatively consistent (Figure 1d: Heteroscedastic Model 2). Overall, our data suggest that transcriptional variation between biological replicates of iPS cells was not substantially, and may be somewhat lower, different from that between passages of an established hES cell line or of adult primary cells.

Given that our variance component analysis was based on FPKM values, we also reanalyzed data excluding highly expressed genes, as this may impact our results. We found that our component estimates and correlation heatmap were qualitatively very similar when the top 1 and 5% of genes were removed from the data set (Figure S7, S8). We also attempted a similar analysis of mitochondrial gene expression. We found similar proportions of reads coming from mitochondrial genes in adult, IPS and ES cells (Figure S9). However, the very low number of expressed genes (13) resulted in extremely noisy estimates of the correlation between sample gene expression profiles (Figure S10) and prohibited variance components analysis, due to failure of the model to converge. Our differential expression analysis classified all mitochondrial genes as “invariant expression” (data not shown). Our experiment did not include multiple replicates from the same cell line, and so we were unable to formally address the issue of variation between lines from the same donor. Visual inspection of our read coverage plots did suggest that some cell lines might be more variable than others. Heatmaps of the differentially expressed genes illustrate that while most cell lines were quite consistent, one cell line derived from keratinocytes formed an outgroup (K-iPSC-S2-1) with other keratinocyte cell lines (Figure S11).

### Cellular memory and aberrant reprogramming are rare in hIPS cells

**Figure 2:**
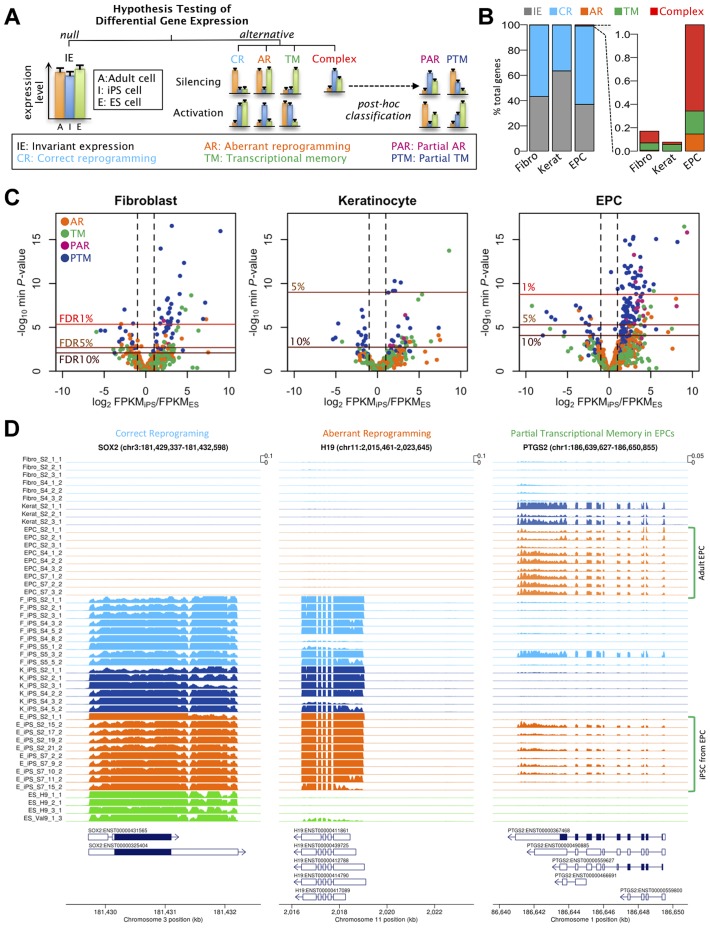
Effects of cell type and tissues of origin on expression levels in iPSCs. (a) Schematic of hypothesis testing approach to identify genes with invariant expression (IE) from genes that we defined as correctly reprogrammed (CR), aberrantly reprogrammed (AR), with transcriptional memory (TM) and showing complex expression. Genes showing complex expression are further classified into partial AR (PAR) and partial TM (PTM) according to the expression patterns. (b) Percentages of genes assigned to alternative differential expression groups based on a hierarchical model (see Text S1 1.4.3). (c) Volcano plots of differentially expressed genes. Plots show log_10_ of minimum P-values among the four alternative hypotheses against log_2_ fold change of average expression levels between iPSCs and ESCs. The colours of points indicate the differential expression categories into which a gene was classified. Dashed lines show twofold enrichment of mean expression levels between iPSCs and ESCs. FDR thresholds were calculated by permutation. (d) Read coverage depth in three examples of differentially expressed genes; SOX2, was categorised as correctly reprogrammed in all three tissues (F-iPSCs: p<1×10^−24^; K-iPSCs: p<1.2×10^−16^;E-iPSCs: p<4.6×10^−25^), H19 was categorised as aberrantly reprogrammed in all three tissues (F-iPSCs: p<1.2×10^−6^; K-iPSCs: p<2.7×10^−4^;E-iPSCs: p<1.5×10^−16^) and TYW3 gene was categorised as partial transcriptional memory in E-iPSCs (p<7.1×10^−8^), invariant expression in F-iPSCs (p>0.1) and correctly reprogrammed in K-iPSCs (p<1.4×10^−3^). Coverage depth was truncated at 1000 reads per bp.

Although our variance components analysis suggested relatively small global effects of tissue of origin, this could mask effects at individual genes. We next sought to identify those genes whose expression in hiPS cells more closely resembled their somatic progenitors or were improperly silenced or activated, relative to ES cells (Fig. 2a). For each of the three somatic tissues in turn, we performed a three-way comparison of expression levels in the somatic tissue, in the hiPS cells derived from that tissue, and in the hES cells. At each gene we tested for departures from a null hypothesis of equal expression levels in the somatic cell, the iPS cells and the ES cells using a negative binomial generalized linear model similar to that outlined in [19] (Text S1). When the null hypothesis (“invariant expression”) was rejected we further classified genes into one of four possible categories, “correctly reprogrammed”, “transcriptional memory”, “aberrantly reprogrammed” and “complex” by selecting the alternative with the highest log likelihood ratio.

We used a hierarchical model (Text S1), similar to that in [20], to estimate the true proportion of transcribed genes in each category without setting a threshold on statistical significance or effect size. Our results suggested that transcriptional memory is very uncommon, occurring at 0.06, 0.06 and 0.20% of all expressed genes in F-, K- and E-iPS cells (Fig 2b). Aberrant or complex expression patterns were also infrequent, although aberrant expression appeared slightly more often in E-iPSCs (0.15% of genes) than in the other two tissues. The fractions falling into the complex category were similarly low (0.10, 0.02 and 0.74% of genes, respectively). The remaining genes either showed invariant expression or were classified as correctly reprogrammed (99.83, 99.92 and 98.92%). Core pluripotency markers, including Sox2, Nanog and Oct4, were all classified as correctly reprogrammed (Figure S12). Our analysis suggested that the fraction of correctly reprogrammed genes is approximately 30-60% of expressed genes. Although comparison between different studies and methodologies difficult, this is not dissimilar to fraction of genes that are differentially expressed in reprogramming found by other studies [21]).

Next we identified individual loci that were differentially expressed at a genome-wide false discovery rate (FDR) of 5% (estimated from permuted data), and that exhibited a 1.5-fold or greater change in expression level between hiPS cells and hES cells. Using these criteria we identified a total of 61, 5 and 103 transcribed regions that showed some form of differential expression in F-, K-, or E-iPS cells respectively (Table 1), the majority of which exhibited either complete or partial transcriptional memory (“TM” or “PTM”). Most genes we identified were very weakly expressed in IPS cells, with mean FPKMs 74–79% lower than the average (Figure S13). The most enriched gene ontology (GO) terms in the TM and PTM gene sets were for mesodermal migration in F-iPS cells (enrichment q-value <0.002;), for hemidesmosome development in K-iPS cells (q<0.03) and for inflammatory and defense response in E-iPS cells (q<3×10^−5^) (Figure S14). Aberrantly expressed genes were less frequent than genes exhibiting transcriptional memory. One interesting exception to this was the long noncoding RNA, H19, which was highly expressed in the majority of hiPS cells in our data set relative to the hES cells (Fig. 2d). Our differential expression analysis also suggested that aberrant activation occurs more often than aberrant silencing (21 versus 6 genes at FDR 5%), and that transcriptional memory more frequently involves memory of an active rather than a silenced gene (101 versus 19 genes at FDR 5%) (Table 1). However, this may simply reflect better power to detect differential expression when a gene is highly expressed rather than silenced. Removal of highly expressed genes had almost no impact on our differential expression analysis (Figures S6, S7). Finally, differential expression analysis of mitochondrial genes classified all 13 genes we found be expressed in any of our samples as “invariant expression”. Overall, our results suggest that, although transcriptional memory and aberrant reprogramming do occur occasionally, relatively few genes are involved, those that are affected are weakly expressed.

**Table 1:** Numbers of significantly (FDR%) differentially expressed genes in each differential expression category.

	AR	PAR	TM	PTM	Total
F-iPSC	9	1	8	43	61
K-iPSC	0	0	1	4	5
E-iPSC	4	15	5	79	103
**Activation**	6	15	9	92	122
**Silencing**	6	0	2	17	25
**Total**	12	15	11	109	147

AR: Aberrant reprogramming; PAR: Partial AR; TM: Transcriptional memory; PTM; Partial TM.

### RNA splicing patterns in hIPS cells resemble ES cells

Although whole gene expression levels appeared to be relatively stable across different tissues of origin, cellular memory could also manifest at the level of RNA splicing. Using the statistical framework we developed for gene expression levels, we next tested whether isoform abundance ratios showed evidence of memory of their cell type of origin (Text S1). In this analysis, we tested the null hypothesis that the ratio of the top two most abundant isoforms was equal in adult tissues, iPS cells and ES cells. We computed abundances using two popular approaches, Cufflinks2 and MISO [22], [23], and analysed the subset of genes where the ranking of isoform abundances agreed between the two methods (Figure S15). We found no significant memory of adult cell splice patterns or aberrant alternative splicing in IPS at an FDR of 5% estimated from permuted data (Figure S16). This suggests that transcriptional memory of cellular splice patterns or aberrant splicing induced during reprogramming is a relatively weak effect, smaller than that observed at the level of whole gene expression, and potentially masked by larger technical and genetic effects.

### Imprinting is conserved between iPS cells and their somatic cell of origin

**Figure 3:**
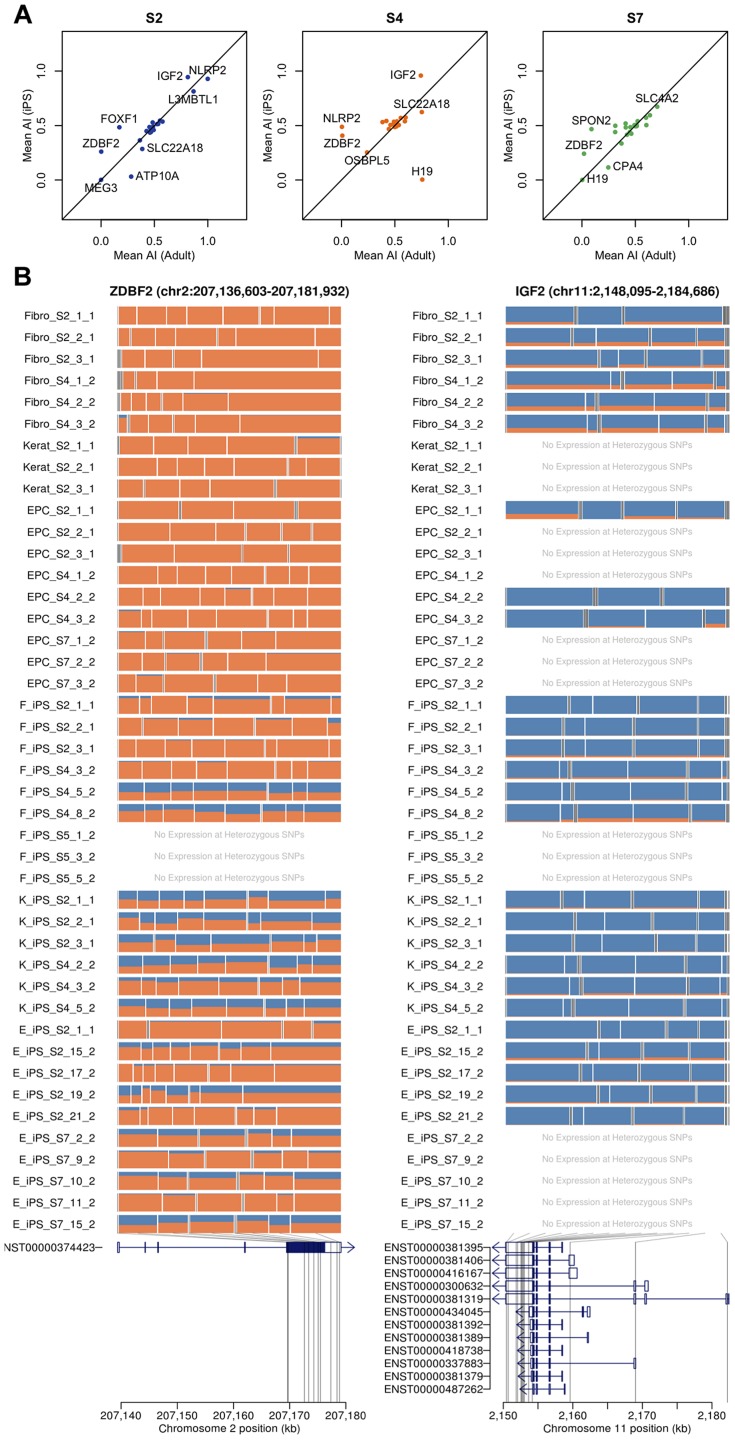
Imprinting effects on transcription in adult and iPS cells. (a) Allelic imbalance between adult cells and iPS cells for each donor (S2, S4 and S7). (b) The mosaic plots for each cell line show the allelic imbalance at multiple heterozygous SNP loci throughout the transcribed region. Each row corresponds to a sample, and each box corresponds to a single heterozygous SNP. Box width is proportional to the total nucleotide count at the heterozygous SNP, with the box bisected in proportion to the paternal and maternal nucleotide counts (blue and orange) based on the phasing haplotype information. Examples show consistent loss of imprinting in iPSCs (ZDBF2) and in adult cells (IFG2).

Previous studies have suggested that imprinted gene expression patterns may be unstable in hES cells and hIPS cells [24]. We genotyped the four individuals from whom our hIPS cell lines were derived using an Illumina Omni2.5 genotyping chip. Genotypes were phased, and SNP genotypes were imputed using Beagle [25]. Using the phased haplotypes, we computed estimates of allele-specific expression for the paternal and maternal chromosomes of each individual. We began by investigating whether patterns of imprinting observed in the adult somatic tissue remained conserved in the iPS cells that were derived from them in a set of 210 putatively imprinted genes obtained from the http://www.geneimprint.com/database. We found that many genes were expressed at a low level or lacked sufficient coverage of heterozygous SNPs, which were subsequently excluded. The remaining genes (26, 23 and 23 in donor S2, S4 and S7, respectively) exhibited allelic expression patterns in adult cells that were conserved in their derived iPS cell lines (Pearson r^2^ = 0.46; p<6.3×10^−10^), Fig 3. Many genes did not demonstrate the characteristic mono-allelic expression of imprinted genes in either adult or pluripotent cells (Fig 3). In a small number of cases, such as the paternally expressed zinc finger gene, ZDBF2, we observed a loss of imprinting and reversion to bi-allelic expression in many IPS lines. We note that, in the cases where we observe loss or alteration of imprinting, the genes involved are relatively weakly expressed in either the adult or the IPS cell, making ascertainment of imprinting status more difficult.

### Detecting known genetic effects on gene expression in hIPSCs

Finally we returned to the effects of the genetic background in our hiPS cells. Extensive maps of genetic variants whose genotype correlates with gene expression (expression quantitative trait loci, eQTLs) have been generated in adult human tissues and cell lines [26]. We investigated whether similar effects could be observed in our iPS cells. Since the number of individuals in our data set was small, a standard eQTL mapping experiment was not possible. Instead, we tested whether genetic associations ascertained in lymphoblastoid cell lines (LCLs), a model system for eQTL detection in humans, were also detectable in iPS cells.

**Figure 4:**
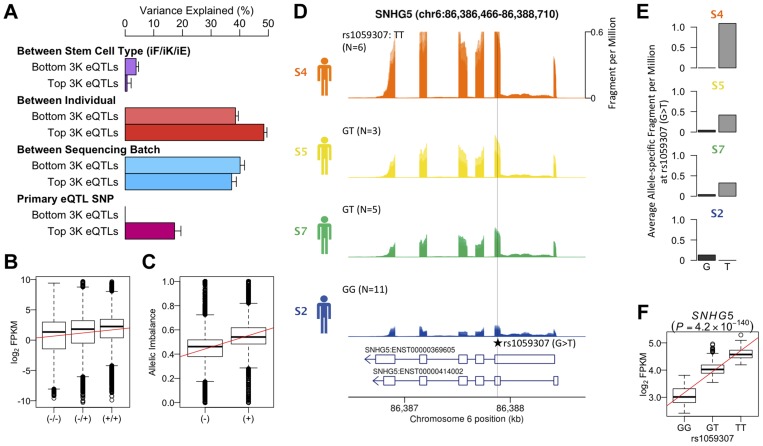
Genetic effects on transcription in iPSCs. (a) Variance component of known eQTL genes showing primary eQTL SNPs being able to explain 17% of FPKM variation on average. Top and bottom 3,000 eQTL genes and SNPs were determined by means of P-values using gEUVADIS RNA-seq data [27]. (b) Box- plot of log2 FPKM aggregated across 462 eQTL genes (FDR 5%) stratified into high (+/+), medium (+/−) and low (−/−) genotypes at the minimum p-value eQTL SNPs ascertained from [2] (FDR 5%). Pearson correlation r = 0.44, p<9.8×10^−68^. (c) Boxplot of allelic imbalance at high-expression (+) and low-expression (−) haplotypes across all eQTL genes ascertained from [2]. The red line is at 0.5 indicating the expected fraction under the null. Student t p<1.3×10^−8^. (d) Mean RNA-Seq coverage depth in iPSCs, averaged across all iPSC lines from each individual, at a putative example eQTL for the noncoding RNA SNHG5 gene. Individuals are grouped by their genotype at the putatively causal variant rs1059307. Individual genotypes are shown with the number of RNA-seq samples in parentheses. (e) Average coverage depth of fragments coming from the high-expression haplotype (allele T) and low- expression haplotype (allele G) at rs1059307. (f) eQTL association of SNHG5 gene at rs1059307 in lymphoblastoid cell lines from [2] showing distributions of log2 FPKM against SNP genotypes at rs1059307. The red line shows the best-fit linear regression line.

We reanalyzed an existing LCL RNA-seq dataset derived from the same source population (162 GBR+CEU individuals) as our hiPS cells (Figure S17) and identified 4,350 eQTLs at an FDR of 5% [27]. Variance component analysis revealed a greater amount of variation between individuals in genes that were ascertained to have an eQTL in LCLs, than in genes where the null of no eQTL could not be rejected (Fig 4a). A substantial fraction (17%) of this variation could be explained by the lead eQTL SNPs (eSNPs). We tested whether the direction of the genetic effect at the LCL eQTL genes replicated in iPS cells by grouping the four individuals in our data set according to their eSNP genotype. We found that the expression level of genes with an eQTL ascertained in LCLs follows the expected direction in hiPS cells (Fig. 4b). Likewise, for individuals in our dataset that are heterozygous at the eSNP we see a corresponding, highly significant allelic imbalance also in the expected direction (Fig. 4c). The correlation between genotype and expression level at ascertained eQTLs in hiPS cells was highly significant (Pearson r = 0.44, p<9.8×10^−68^), as was the allelic imbalance at heterozygous ascertained eSNPs (Student t, p<1.3×10^−8^). Although our data set is small, we do find convincing examples where a correlation between genotype and gene expression replicated a known eQTL identified in LCLs such as the exonic eSNP, rs1059307, located within the noncoding RNA gene SNHG5. At this gene, we also observe clear allelic imbalance in iPS cells derived from S5 and S7 individuals, who are heterozygous for this eSNP (Fig. 4e), which is in the same direction as the eQTL effect in LCLs (Fig. 4f). These genetic effects on gene expression in iPS cells were detectable across multiple independent iPS cells from the same genetic background, despite the variety of different tissues sources and reprogramming methods.

## Discussion

We have shown that epigenetic memory of the adult progenitor cell is a rare phenomenon in hIPS cells, and that cellular heterogeneity between different hIPS lines is more likely to be driven by changing genetic background. Our study has important implications for future attempts to use iPSCs as cellular model systems for drug discovery and other applications. Encouragingly, our results suggest that genetic effects are readily detected in hIPSCs and that cell phenotypes are highly reproducible within individuals. Equally important, however, is the fact that the noise introduced by genetic background could potentially obscure small genetic signals of interest in small samples. A clear implication of this result is that, in iPSC-based studies of genetic disease, most effort should be expended on collection of samples from different donors rather than generation of large numbers of lines from the same individual. Collection of multiple individuals, perhaps with a shared genotype at a single locus of interest, will allow the effects of genetic background to be averaged over and separated from that of the putatively causal locus.

Our study also highlights how the effects of genetic background cannot be ignored when considering cellular variability between pluripotent stem cell lines. Previous studies have attributed cellular variability in IPS to a range of sources, including epigenetic memory [5]–[10], [17], inherent differences between IPSCs and ESCs [4], [28], artifacts of reprogramming [7] or lab environment [29]. Perhaps surprisingly, the effects of genetic background have been less well appreciated, although more recent work has highlighted its potential importance in differentiation [30]. It seems likely that at least some of variability previously reported to exist in IPS cells could in fact have arisen from genetic differences. This is particularly true of comparisons of IPS with ES that are typically derived from different individuals. It is also notable that studies including larger numbers of donors tend to find fewer transcriptional differences between IPS and ES [29], [31]–[33]. Studies that have not controlled for genetic background when investigating epigenetic memory, such as by confounding tissue of origin and donor, may also have mistakenly attributed genetically driven differences in transcription to epigenetic memory. Our study explicitly incorporates multiple tissues from the same donors, allowing us to correct for the effects of changing genetic background. This is likely to explain why we do not find extensive apparent epigenetic memory. We note, however, that other studies that have also explicitly controlled for genetic background still report some variation in transcription, methylation and differentiation efficiencies that appear to arise from cell type of origin effects [5], [8], [10]. A possible explanation for the discrepancy between the results of these studies and our own is that we have also taken our samples from cells at between 10 and 13 passages and epigenetic memory effects may be transient and disappear following multiple passages [8].

An important caveat for our study is that influential cellular differences may simply not manifest as transcriptional variation but reside at, for example, the epigenetic level as changes in methylation status or histone tail modifications. Such differences may harbor a “hidden” functional role that only becomes apparent upon differentiation into a specific cell lineage. Our study suggests that epigenetic differences are likely to be more plausible candidates as drivers of variation in IPS cell differentiation ability. However, our results also illustrate that current iPS cell technology is robust enough to enable detection of genetic effects on important cellular phenotypes such as mRNA levels. Although further technological hurdles remain, an exciting area for future work will be detection of regulatory variation that influences transcription during cell lineage specification and differentiation, employing iPS cells as a model system.

## Materials and Methods

### Samples

All primary tissue samples and blood for this project were obtained from adult cadaveric organ transplant donors referred to the Eastern Organ Donation Services Team (part of NHS Blood and Transplant). Ethics approval was obtained from the local Research Ethics Committee (REC No. 09/H306/73).

### Derivation of fibroblasts and keratinocytes from skin samples

For each subject included in this study, a sample of skin was excised from the midline surgical incision. The skin was transported to the lab and washed in iodine and ethanol and was cut into approximately 1 mm^3^ pieces. These were dispersed evenly on a 90 mm plate and incubated with fibroblast media (Knockout DMEM and 10% FBS). Outgrowths of fibroblasts and keratinocytes from the skin explants were usually apparent at around 14 days. The cells were separately harvested using 5 min treatment with Versene (15040-066, Invitrogen), which detached the fibroblasts leaving the keratinocytes on the plate. The fibroblasts were cultured on non-coated plates using fibroblast media and keratinocytes were cultured on plates coated with matrix (R011K, Invitrogen) and using EpiLife media plus Defined Growth Supplement (M-EPI-500-CA and S-012-5, Invitrogen).

### Derivation of Endothelial Progenitor Cells (EPCs)

Endothelial Progenitor Cells were derived from 100 mL of peripheral blood as previously described [11]. Briefly, the mononuclear cells of the blood sample were separated using Ficoll. The cells were cultured on collagen-coated plates using EPC media (EGM-2MV supplemented with growth factors plus 20% Hyclone serum; CC-3202, Lonza and HYC-001-331G; Thermo Scientific Hyclone respectively). Colonies of EPCs appeared at around 10 days.

### Generation of EPC-hiPSCs using retroviruses

Four pseudotyped Moloney murine leukemia retroviruses containing the coding sequences each of human OCT-4, SOX-2, KLF-4 and C-MYC were obtained from Vectalys (Toulouse, France). A multiplicity of infection of 10 was used in all retroviral reprogramming experiments. For each hiPS cell derivation, 1×10^5^ EPCs were transfected with the 4 viruses in the presence of 10 ug/mL of polybrene (TR-1003-G, Millipore). After 24 hrs the viruses were washed off with PBS and the cells were re-fed with EPC media that remained for the next 4 days. On day 5 after transduction, the cells were re-plated using trypsin onto a 10 cm dish of fresh MEF feeders. After 2 days the media was changed from primary cell-specific to hiPSC media (KSR + FGF-2). The media was changed every 2 days until colonies emerged after which the media was changed daily. Colonies were picked once they had reached sufficient size, typically from day 25 following transduction. The colony was split into quarters and the segments gently lifted off the plate and transferred to one well of a 12 well plate of fresh MEF feeders containing hiPS cell media (KSR + FGF2) supplemented with ROCK inhibitor (Y-27632, Sigma).

### Generation of F-hiPS cells and K-hiPS cells using Sendai virus

Four Sendai viruses containing the coding sequences of each of human OCT-4, SOX-2, KLF-4 and C-MYC were obtained from DNAVec (Ibaraki, Japan). The protocol for reprogramming was identical to that of retroviruses with the exceptions that 5×10^5^ primary cells (fibroblasts or keratinocytes) were used at MOI 3 and polybrene was not used.

### hiPS cell culture

hiPS cells were grown on irradiated MEF feeders, using human embryonic stem cell media (termed KSR + FGF-2): Advanced DMEM (12634-010, Invitrogen) was supplemented a follows: 10% Knockout Serum Replacement (10828028, Invitrogen), 2 mM L-glutamine (25030024, Invitrogen, 0.1 mM β-mercaptoethanol (M6250, Sigma-Aldrich) and 4 ng/µL of recombinant human basic Fibroblast Growth Factor-2 (233-FB-025, R&D systems, Minneapolis, MO, USA). Media was changed daily and the cells were passaged every 5–10 days depending on the confluence of the plates. To passage hiPS cells, the plates were washed in PBS and colonies detached using collagenase and dispase (Collagenase IV 1 mg/mL, Invitrogen 17104-019; Dispase 1 mg/mL, Invitrogen 17105-041). The colonies were washed in media and mechanically broken up before being re-plated onto fresh MEF feeders.

### RNA extraction

Total RNA was extracted using the RNeasy Mini Kit protocol (Qiagen, Hilden, Germany). RNA-seq libraries were constructed according the manufacturers guidelines, with minor modifications, using the Illumina mRNA-seq and TruSeq mRNA sample preparation kits (Illumina, Inc., San Diego, CA). Briefly, mRNA was enriched from total RNA using oligo dT beads before fragmentation via zinc and heat hydrolysis. mRNA was subject to first and second strand cDNA synthesis before end repairing and A-tailing. Double-stranded cDNA was then adapter-ligated before size-selecting fragments with inserts ranging from 200–300 bp using a LabChipR XT (Perkin Elmer, Waltham, MA). Size-selected material was then PCR-amplified using KAPA HiFi polymerase (Kapa Biosystems, Boston, MA) before sequencing on an Illumina HiSeq2000 (Illumina, Inc., San Diego, CA).

### Computational and statistical analysis

We mapped reads to assembly h37 of the human genome using Bowtie2 [14] and constructed spliced alignments using Tophat2 [34] with default settings. We also used known gene annotation information given by Ensembl release 69 as a guide for the alignment. Following read mapping, we selected fragments (read-pairs) where at least one of mate-pairs had a quality score of >10, aligned with no gaps, with three base mismatches or less. Any read pairs with an insert size less than 150 bp or greater than 1 Mb, or on different chromosomes, were excluded from subsequent analyses. Computational analysis was carried out using a combination of existing packages, such as DESeq [19] and our own analysis tools. For the variance components analysis, transcription level at each gene *j* was modeled as a linear combination of five normally distributed random effects (*b*_1–5_) and a single error term:
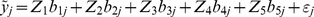
where 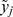 is a vector of log normalized fragments per kilobase per million reads sequenced (FPKMs) for gene *j* in each of the 46 samples in our data set, *b*_1_ is an intercept term, *b*_2_ models variation in transcription between the three adult somatic tissues, hESCs and hiPSCs, *b*_3_ models differences between F-, K- and E-iPSCs, *b*_4_ captures transcriptional variation between different donors, *b*_5_ captures differences between the two sequencing batches in our data set, ε is the error term and *Z*_1_-*Z*_5_ are design matrices. For full details of computational and statistical analyses, see Text S1.

### Data

Our raw sequence data are available from the European Genotype Archive under study ID EGAS00001000367. A variety of processed data, including raw read counts, log2 FPKMS and the results of our differential expression analysis are available from our lab website (http://www.sanger.ac.uk/research/projects/genomicsofgeneregulation/) under the “Data” tab.

## Supporting Information

Figure S1 Differentiation of iPSCs to endo-, meso- and neuroectoderm. hIPSCs generated using Sendai Virus can differentiate into cells expressing markers specific of the three germ layers. hIPSCs (S5SF5) were differentiated into neuroctoderm, endoderm and mesoderm using defined culture conditions as described previously [13]. The resulting cells were analysed for the expression of specific germ layers markers using immunostaining. Blue fluorescence shows DAPI staining. Similar results were obtained with other hIPSCs lines used for this study. Scale bar 100 µM.
(JPG)

Figure S2 RT-PCR for Sendai viral genome and transgenes in a subset of lines. Gels show results of RT-PCR using viral primer sets as described in the CytoTune-iPS reprogramming kit (Invitrogen) in line S4SK4 (passage 3). Results are shown are for Sendai virus genome (SeV: 181 bp amplicon), Sendai-derived exogenous Oct3/4 (O: 483 bp amplicon), Sox2 (S: 451 bp am- plicon), Klf4 (K: 410 bp amplicon) and cMyc (M: 532 bp amplicon).
(PDF)

Figure S3 The number of sequenced reads for each sample. Polyadenylated RNA was extracted from each cell culture and multiplexed cDNA libraries were synthesized. For each sample, we performed 75 bp paired end sequencing on the Illumina HiSeq2000 platform. In total we generated 7.3 billion reads, with between 85.3 and 229.8 million reads sequenced in each sample.
(PNG)

Figure S4 The number of reads mapped onto the reference genome for each sample. We mapped reads to assembly h37 of the human genome using Bowtie2 and constructed spliced alignments using Tophat2. Following read alignment and QC filtering, between 49% and 89% of reads mapped uniquely to the human genome.
(PNG)

Figure S5 Distribution of FPKMs in adult, IPS and ESCs. Distribution of log10 (FPKM+1) for all known protein coding genes from ENSEMBL. Each line shows the distribution for a single sample, with the heavier line showing the mean for each cell type. Inset shows the probability that gene is classified as coming from the low/repressed mode of the FPKM distribution estimated using a two component Gaussian mixture model to classify genes into active or repressed. Left panel shows distribution for all genes, right panel excluding the top 1% expression genes.
(PDF)

Figure S6 Percentage reads mapping to LINE and LTRs elements Bars show the percentage of total mapped reads that map to LINE and LTR repetitive elements outside known transcribed regions as annotated in the UCSC repetitive elements track. Blue denotes adult cells, orange denotes IPS cells and green denotes ESCs.
(PDF)

Figure S7 Variance component analysis and differential expression (DE) analysis excluding highly expressed genes (upper 1%-tile). (a) Correlation heatmap without upper 1%-tile highly expressed genes (b) Result of variance component analysis without upper 1%-tile highly expressed genes. (c) P-value comparison with original DE analysis. Each panel shows scatter plot of the DE minimum P-values without upper 1%ile highly expressed genes (X-axis) against original minimum DE P-values (Y-axis) for each tissue. Gray vertical and horizontal lines show 5% FDR.
(PDF)

Figure S8 Variance component analysis and differential expression (DE) analysis with genes without highly expressed genes (upper 5%-tile). (a) Correlation heatmap without upper 5%-tile highly expressed genes (b) Result of variance component analysis without upper 5%-tile highly expressed genes. (c) P-value comparison with original DE analysis. Each panel shows scatter plot of the DE minimum P-values without upper 5%-tile highly expressed genes (X-axis) against original minimum DE P-values (Y-axis) for each tissue. Gray vertical and horizontal lines show 5% FDR.
(PDF)

Figure S9 Percentage of total fragments mapping to 13 mitochondrial protein coding genes. Bars show the percentage of total reads mapping to known mitochondrial genes in all samples in our data. Blue denotes adult cells, orange denotes IPS cells and green denotes ESCs.
(PDF)

Figure S10 Mitochondrial gene expression. Correlation heatmap of log2 FPKMs for 13 mitochondrial protein coding genes. Map elements show Spearman correlation coefficients.
(PDF)

Figure S11 Heatmaps of log2 FPKMs for partial transcriptional memory (PTM) genes deter- mined by the differential expression analysis. (a) Partial transcriptional memory genes in F- iPSCs, (b) K-iPSCs and (c) E-iPSCs. We note that, although patterns of expression across most lines are broadly consistent with one another, line K-iPSC-S2-1-1 forms an outlier from the other K-iPSCs
(PDF)

Figure S12 Coverage depth plots of core pluripotency marker genes. Plots show read coverage of three core pluripotency markers, SOX2, NANOG and OCT4 from left to right.
(PDF)

Figure S13 Mean expression levels of differentially expressed genes. Plots show the densities of log_10_(FPKM) in all genes (black lines) and in genes that were detected as differentially expressed (DE; either transcriptional memory, or aberrant reprogramming; red lines) in our analysis.
(PNG)

Figure S14 Coverage depth plots of genes driving Gene Ontology enrichments in F- and K-iPS cells. Plots show coverage depth for four genes, KRT5, KRT14, MESP1 and MESP2, that were annotated with the most significant Gene Ontology term enrichments (“hemidesmosome assembly” and “mesoderm migration involved in gastrulation”) in K-iPS cells and F-iPS cells, respectively. Coverage depth was truncated at 500 reads per bp.
(PDF)

Figure S15 Scatterplot of transcript FPKMs between MISO and Cufflinks. Plotted is the distribution of FPKMs of all known annotated transcripts estimated by Cufflinks (X-axis) against MISO (Y-axis). Overall, the FPKM estimation is consistent so that many transcripts are seen on the diagonal line. However, there are also a certain amount of transcripts only enriched in one of the two methods.
(JPG)

Figure S16 Volcano plots of differential isoform expression. Plots show log_10_ of minimum P-values among the four alternative hypotheses against the maximum log_2_ fold-change of average transcript expression levels between iPSCs and ESCs. The colours of the points indicate the differential expression categories into which a gene was classified. Dashed lines show twofold enrichment of mean expression levels between iPSCs and ESCs. The FDR threshold was calculated by the permutation scheme as in the differential expression analysis.
(PNG)

Figure S17 Result of population stratification for our samples (S2/S4/S5/S9) with 1000 Genomes Project data. Principal component analysis was performed using Eigenstrat [35] with genome-wide SNP genotypes of European populations obtained from 1000 Genomes Project. All four samples are clustered with GBR and CEU populations.
(PNG)

Figure S18 GC content influences inference of expression levels from RNA-seq. Plotted is the log_2_ relative enrichment, *F_il_*, against the mean GC content of bin *l* for all samples. The red line shows the fitted spline function, *F_il_* (see Text S1 for details).
(JPG)

Figure S19 Relationship between precision and expression level. Plotted is the distribution of log τj against average of log2 normalised FPKMs y ˜j across all samples (j = 1,…, L).
(PNG)

Text S1 Supplementary methods.
(PDF)
