# Supplementary figures and images for: Genetic Background Drives Transcriptional Variation in Human Induced Pluripotent Stem Cells
Source: PLoS Genet. 2014 Jun 5;10(6):e1004432. doi: 10.1371/journal.pgen.1004432 (PMC4046971; doi:10.1371/journal.pgen.1004432)

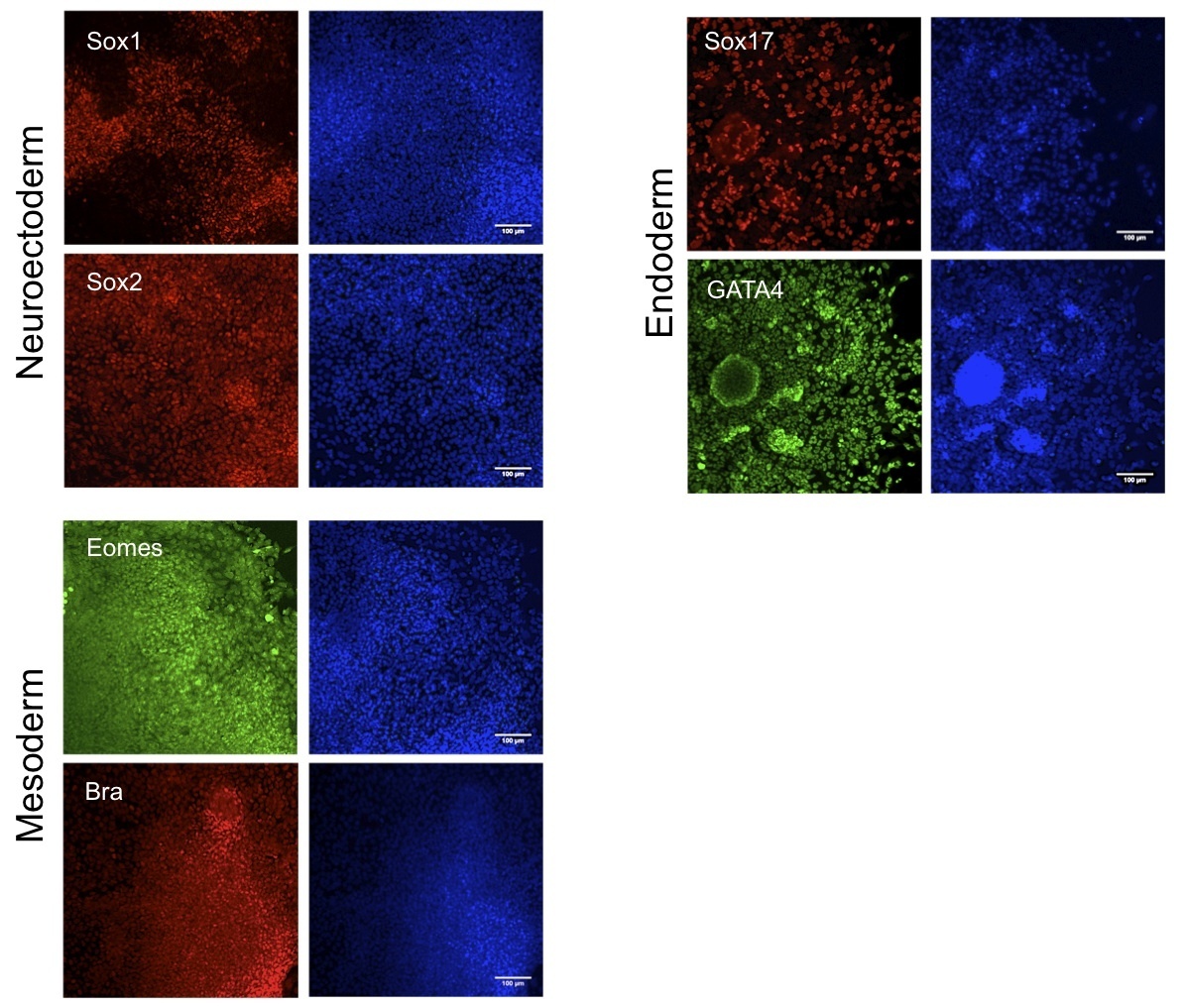

Supplement: Figure S1 — Differentiation of iPSCs to endo-, meso- and neuroectoderm. hIPSCs generated using Sendai Virus can differentiate into cells expressing markers specific of the three germ layers. hIPSCs (S5SF5) were differentiated into neuroctoderm, endoderm and mesoderm using defined culture conditions as described previously [13]. The resulting cells were analysed for the expression of specific germ layers markers using immunostaining. Blue fluorescence shows DAPI staining. Similar results were obtained with other hIPSCs lines used for this study. Scale bar 100 µM. (JPG) [file pgen.1004432.s001.jpg]

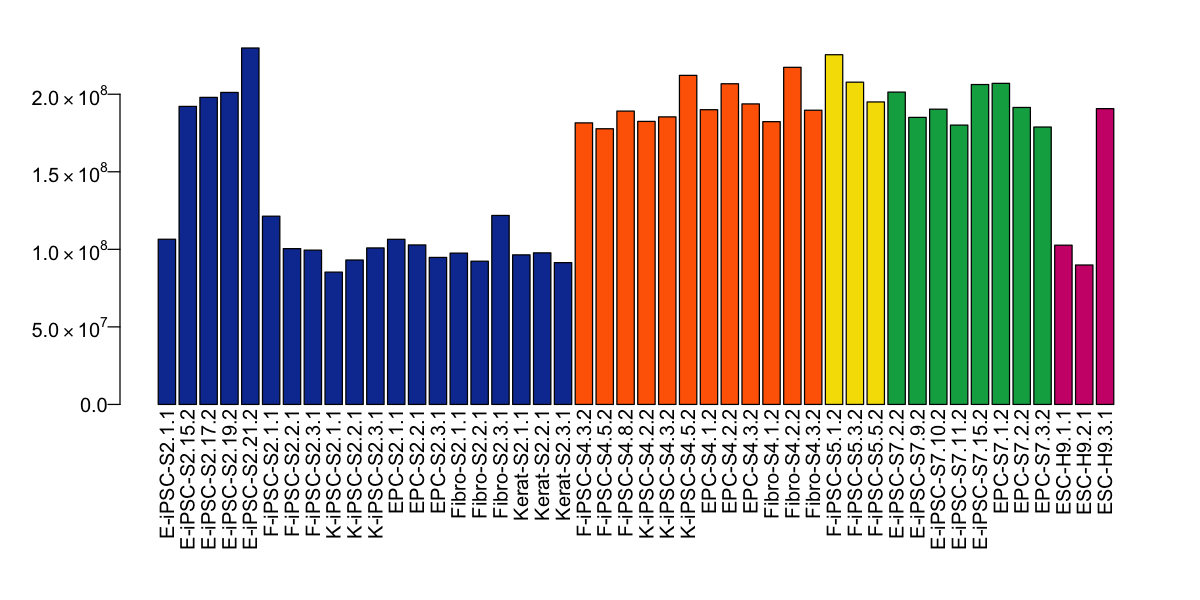

Supplement: Figure S3 — The number of sequenced reads for each sample. Polyadenylated RNA was extracted from each cell culture and multiplexed cDNA libraries were synthesized. For each sample, we performed 75 bp paired end sequencing on the Illumina HiSeq2000 platform. In total we generated 7.3 billion reads, with between 85.3 and 229.8 million reads sequenced in each sample. (PNG) [file pgen.1004432.s003.png]

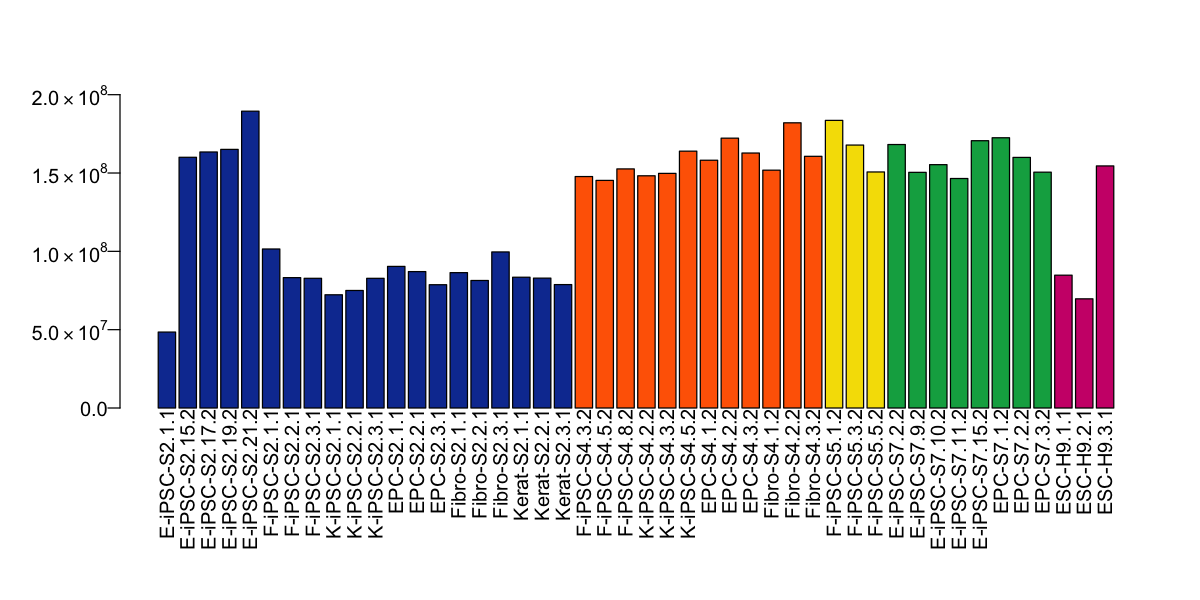

Supplement: Figure S4 — The number of reads mapped onto the reference genome for each sample. We mapped reads to assembly h37 of the human genome using Bowtie2 and constructed spliced alignments using Tophat2. Following read alignment and QC filtering, between 49% and 89% of reads mapped uniquely to the human genome. (PNG) [file pgen.1004432.s004.png]

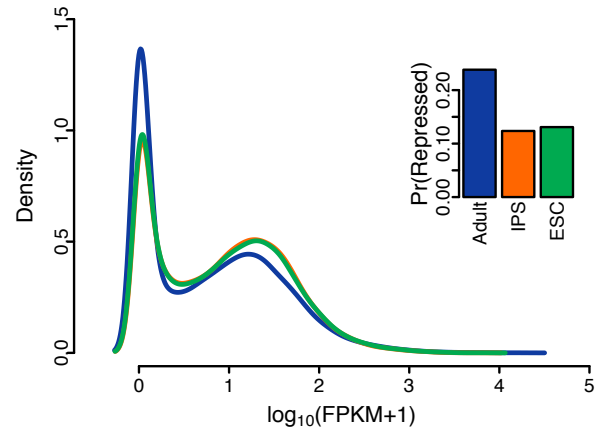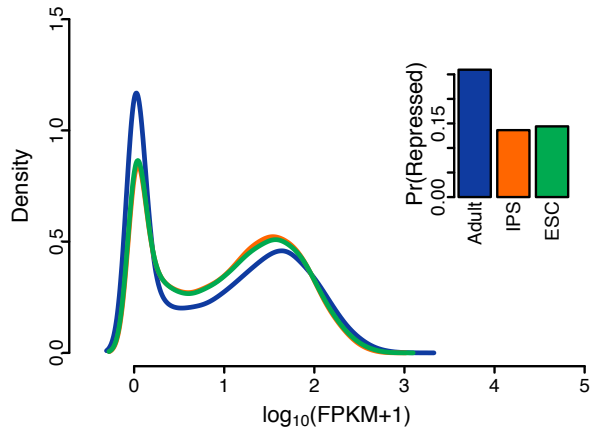

Supplement: Figure S5 — Distribution of FPKMs in adult, IPS and ESCs. Distribution of log10 (FPKM+1) for all known protein coding genes from ENSEMBL. Each line shows the distribution for a single sample, with the heavier line showing the mean for each cell type. Inset shows the probability that gene is classified as coming from the low/repressed mode of the FPKM distribution estimated using a two component Gaussian mixture model to classify genes into active or repressed. Left panel shows distribution for all genes, right panel excluding the top 1% expression genes. (PDF) [file pgen.1004432.s005.pdf]

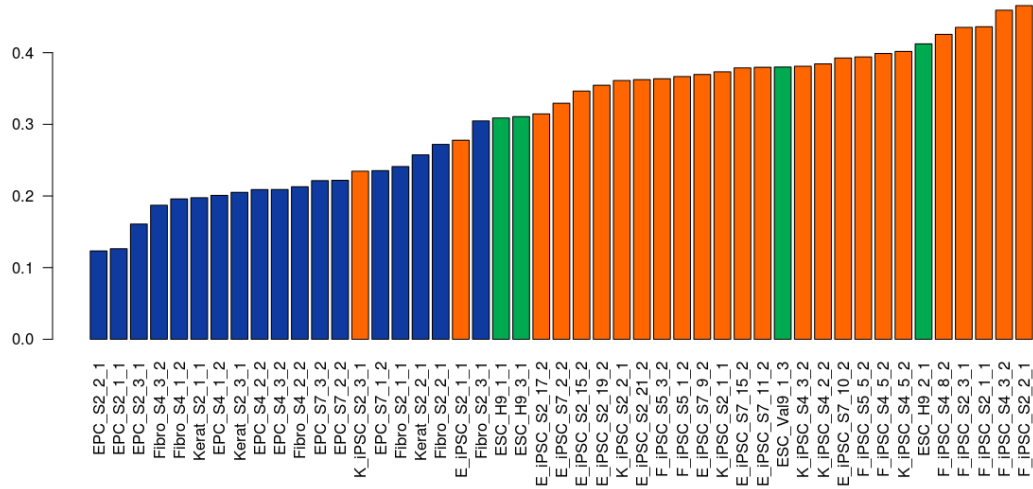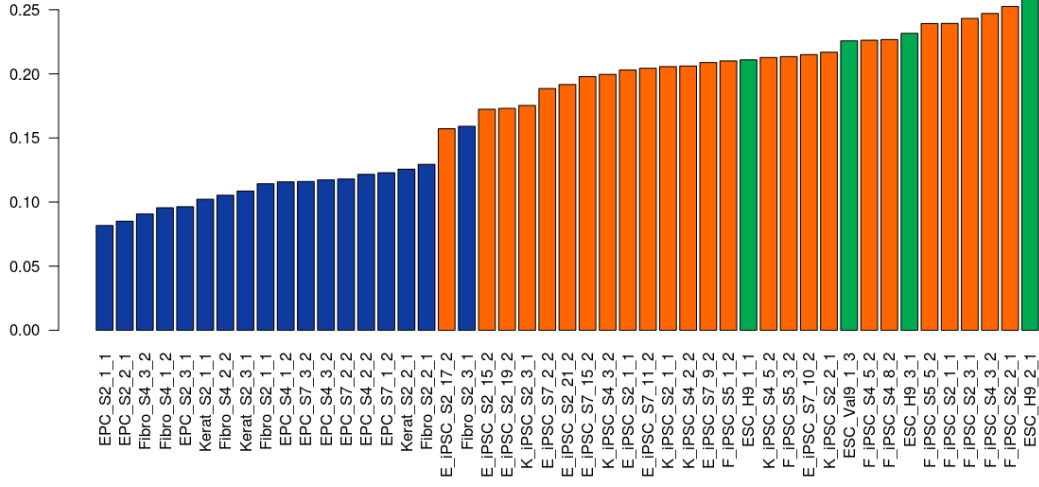

Supplement: Figure S6 — Percentage reads mapping to LINE and LTRs elements Bars show the percentage of total mapped reads that map to LINE and LTR repetitive elements outside known transcribed regions as annotated in the UCSC repetitive elements track. Blue denotes adult cells, orange denotes IPS cells and green denotes ESCs. (PDF) [file pgen.1004432.s006.pdf]

**a**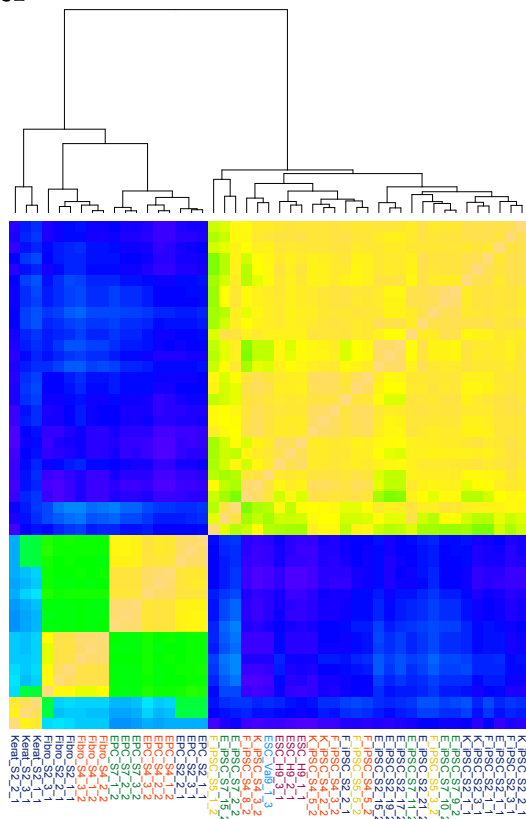**b**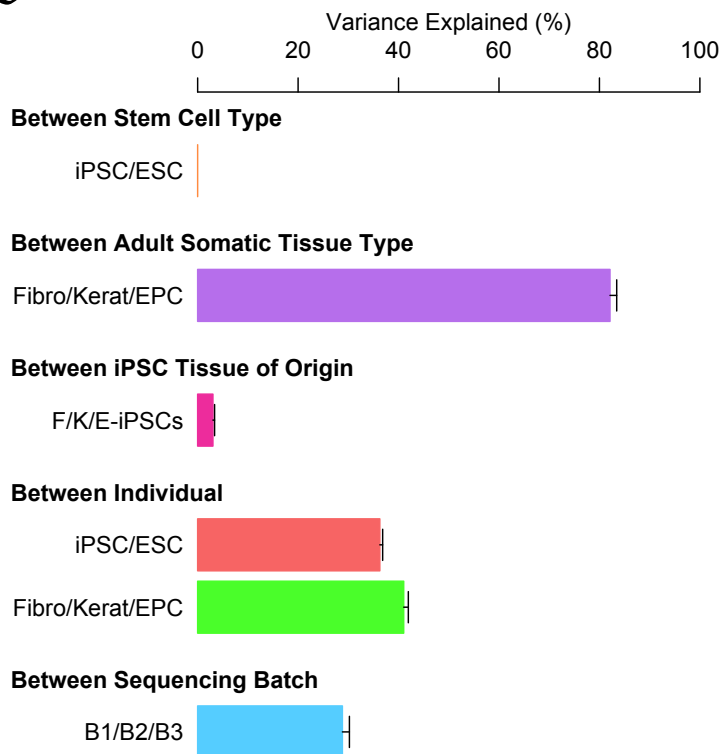**c**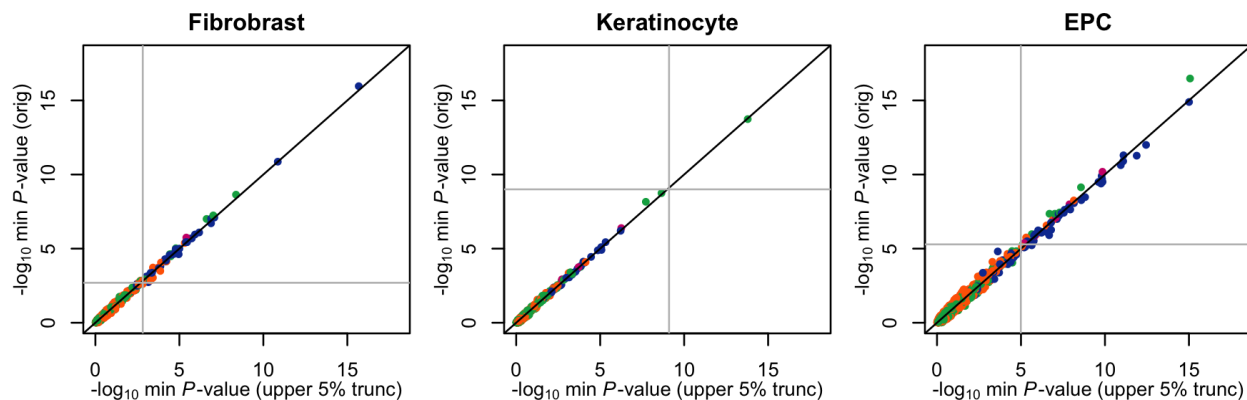

Supplement: Figure S8 — Variance component analysis and differential expression (DE) analysis with genes without highly expressed genes (upper 5%-tile). (a) Correlation heatmap without upper 5%-tile highly expressed genes (b) Result of variance component analysis without upper 5%-tile highly expressed genes. (c) P-value comparison with original DE analysis. Each panel shows scatter plot of the DE minimum P-values without upper 5%-tile highly expressed genes (X-axis) against original minimum DE P-values (Y-axis) for each tissue. Gray vertical and horizontal lines show 5% FDR. (PDF) [file pgen.1004432.s008.pdf]

MT fragment %

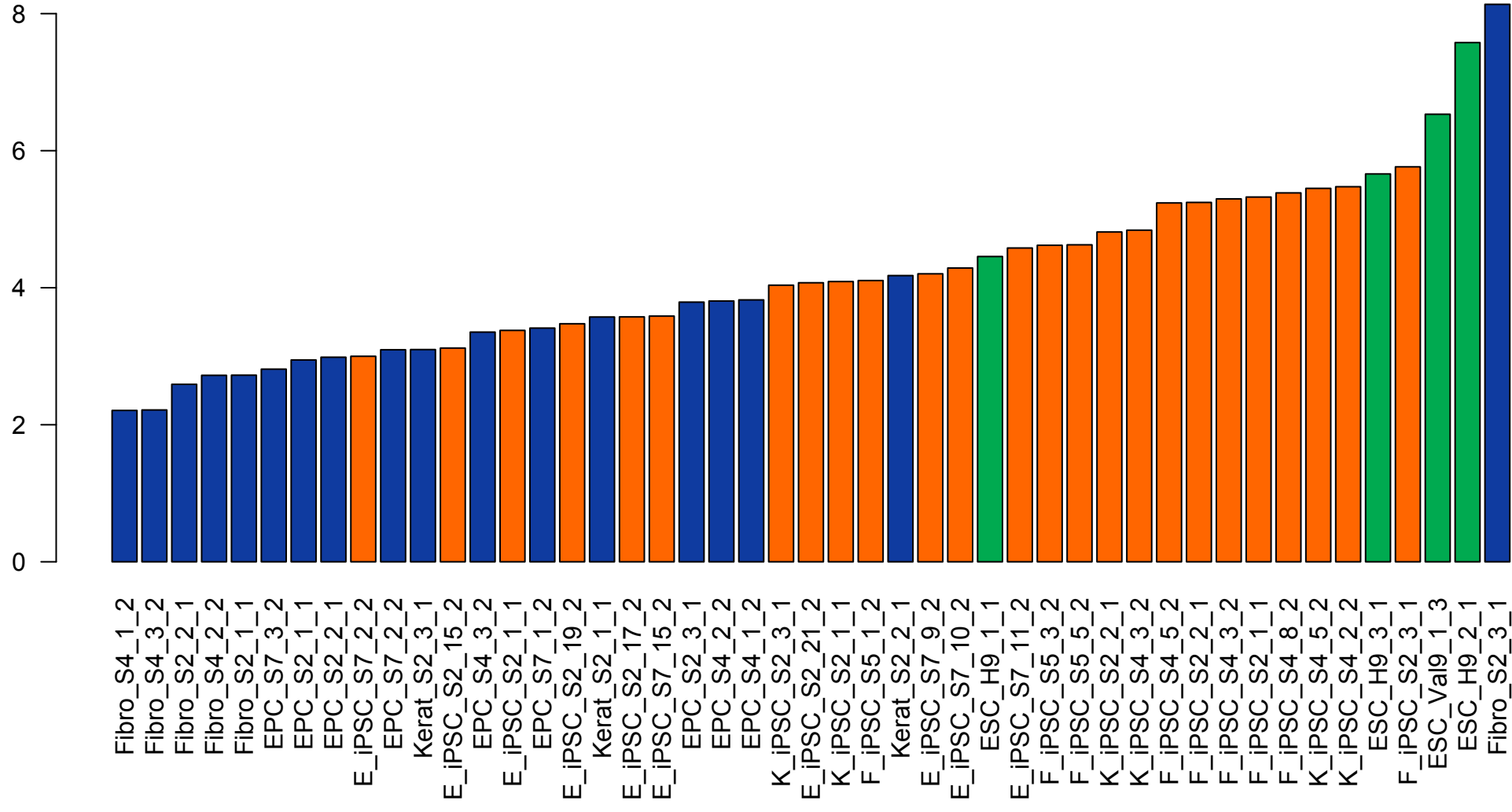

Supplement: Figure S9 — Percentage of total fragments mapping to 13 mitochondrial protein coding genes. Bars show the percentage of total reads mapping to known mitochondrial genes in all samples in our data. Blue denotes adult cells, orange denotes IPS cells and green denotes ESCs. (PDF) [file pgen.1004432.s009.pdf]

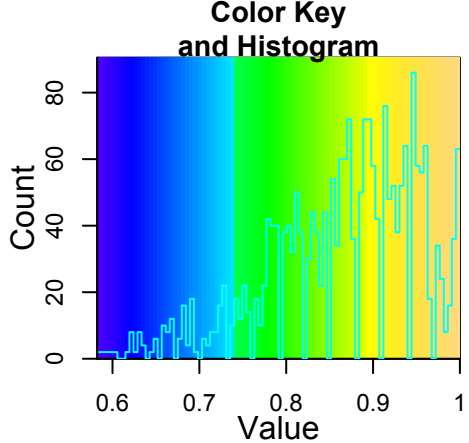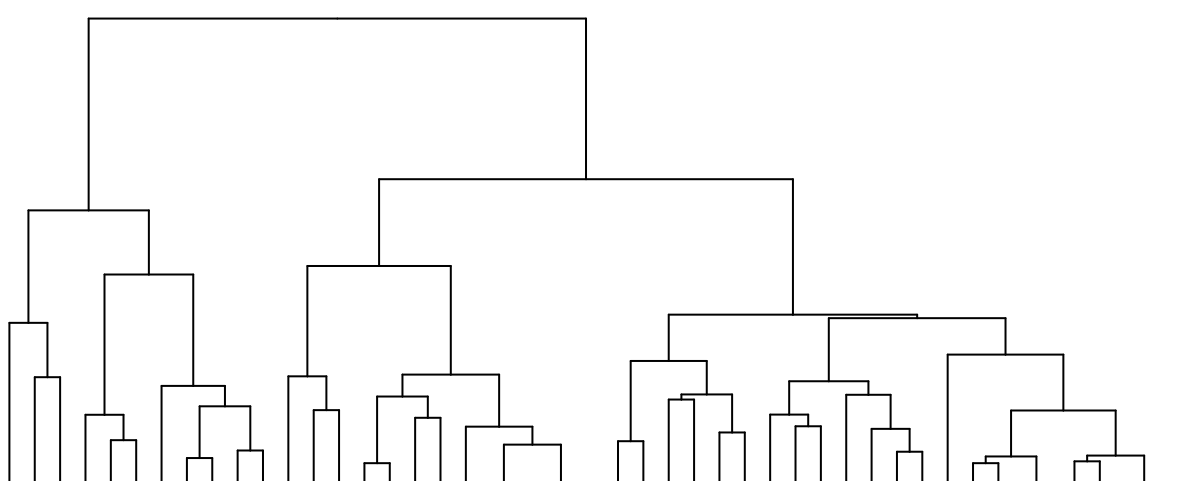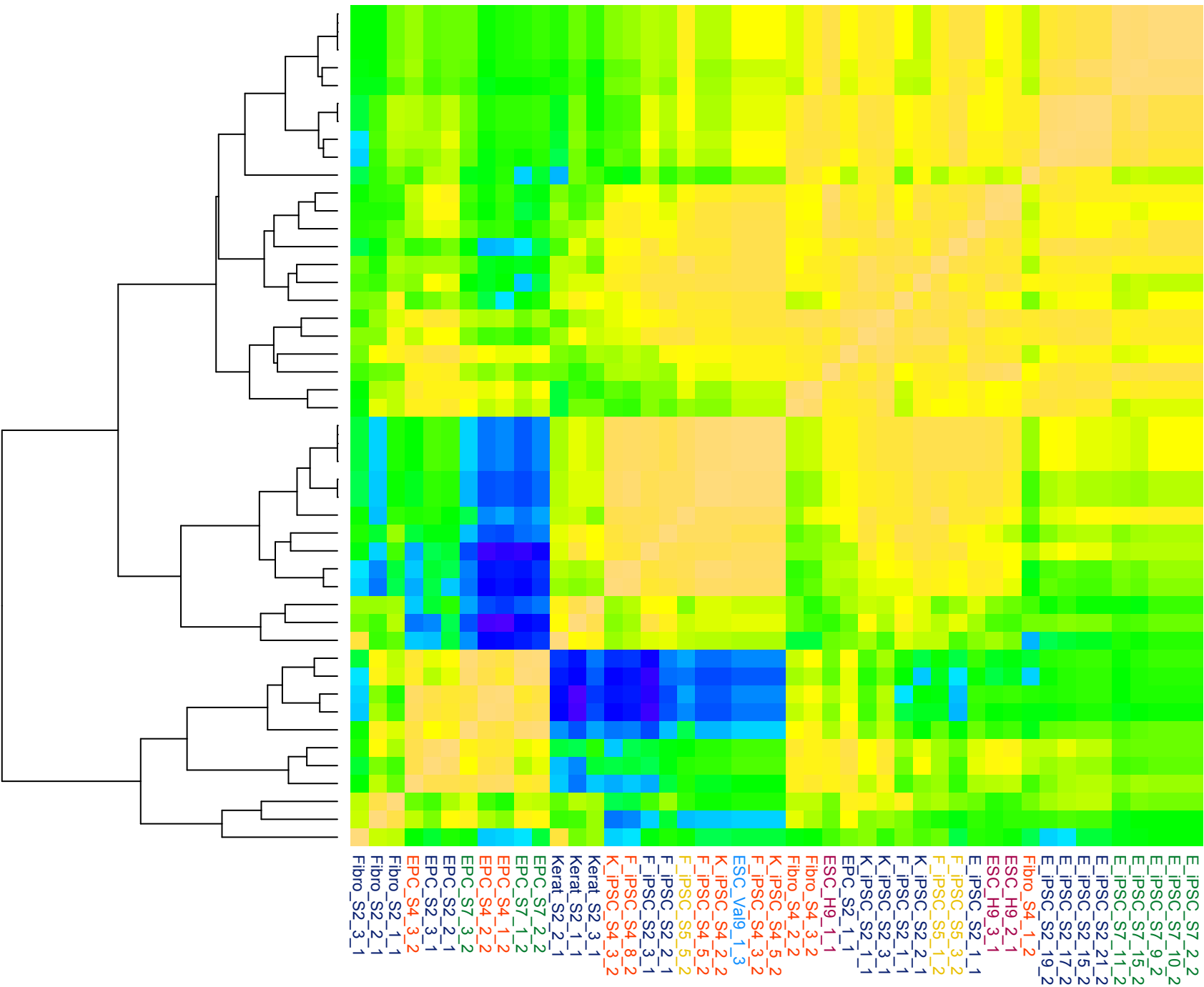

Supplement: Figure S10 — Mitochondrial gene expression. Correlation heatmap of log2 FPKMs for 13 mitochondrial protein coding genes. Map elements show Spearman correlation coefficients. (PDF) [file pgen.1004432.s010.pdf]

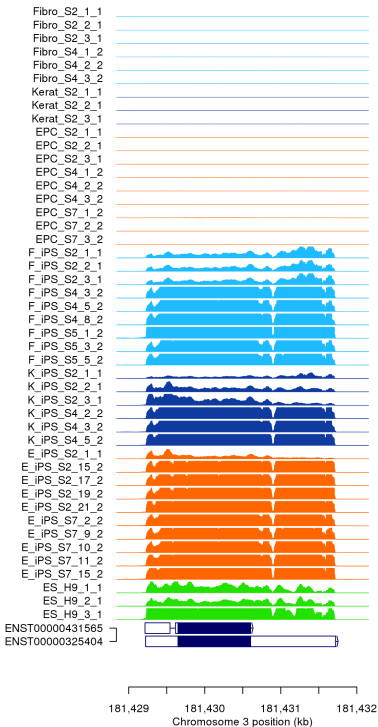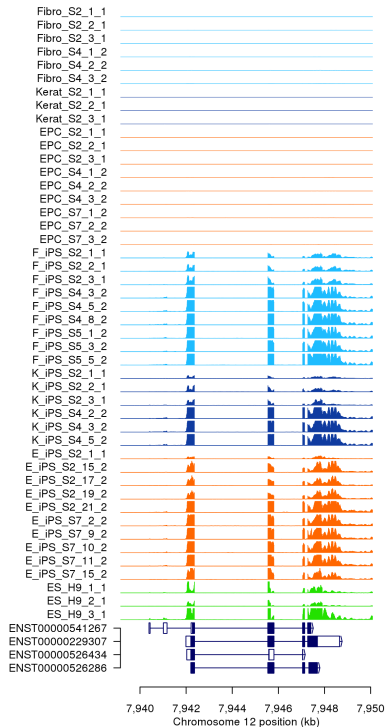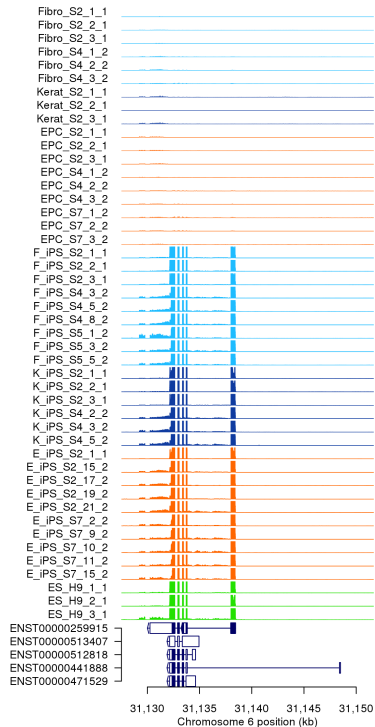

Supplement: Figure S12 — Coverage depth plots of core pluripotency marker genes. Plots show read coverage of three core pluripotency markers, SOX2, NANOG and OCT4 from left to right. (PDF) [file pgen.1004432.s012.pdf]

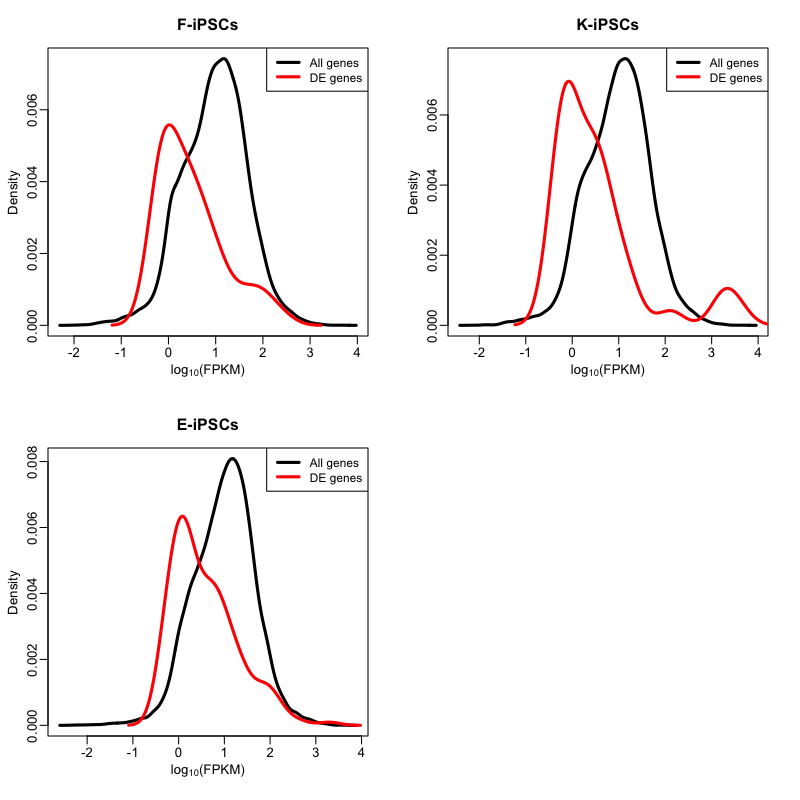

Supplement: Figure S13 — Mean expression levels of differentially expressed genes. Plots show the densities of log10(FPKM) in all genes (black lines) and in genes that were detected as differentially expressed (DE; either transcriptional memory, or aberrant reprogramming; red lines) in our analysis. (PNG) [file pgen.1004432.s013.png]

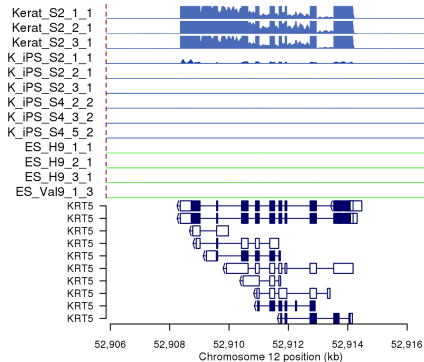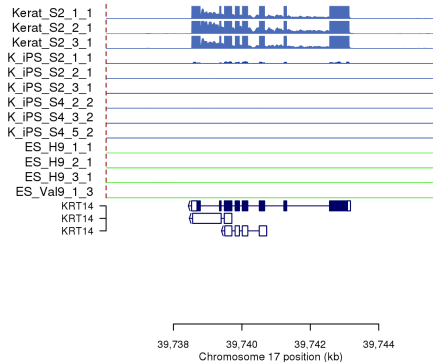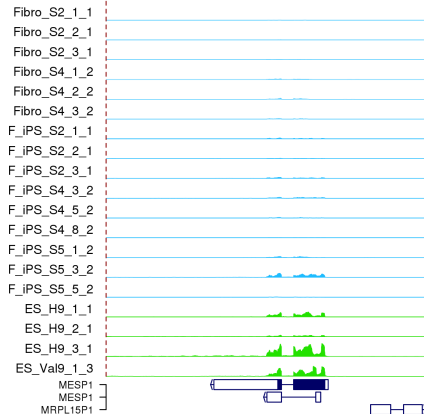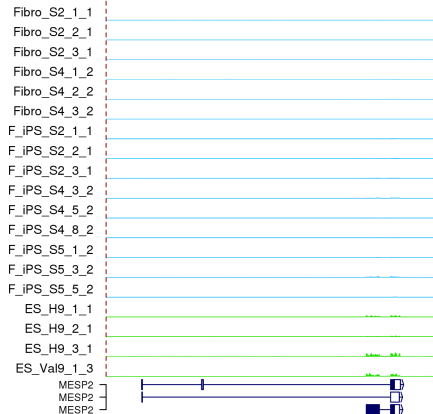

Supplement: Figure S14 — Coverage depth plots of genes driving Gene Ontology enrichments in F- and K-iPS cells. Plots show coverage depth for four genes, KRT5, KRT14, MESP1 and MESP2, that were annotated with the most significant Gene Ontology term enrichments (“hemidesmosome assembly” and “mesoderm migration involved in gastrulation”) in K-iPS cells and F-iPS cells, respectively. Coverage depth was truncated at 500 reads per bp. (PDF) [file pgen.1004432.s014.pdf]

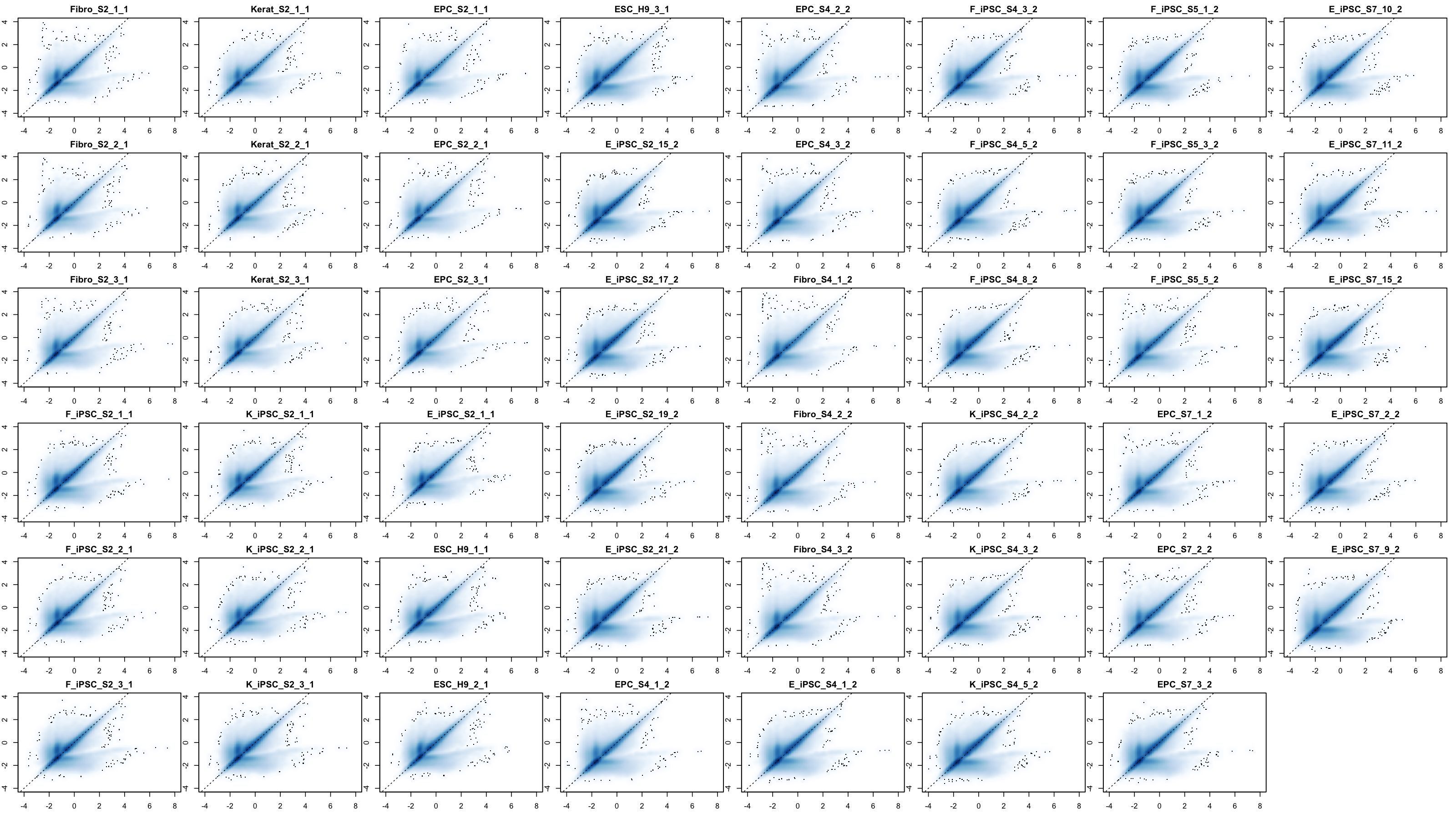

Supplement: Figure S15 — Scatterplot of transcript FPKMs between MISO and Cufflinks. Plotted is the distribution of FPKMs of all known annotated transcripts estimated by Cufflinks (X-axis) against MISO (Y-axis). Overall, the FPKM estimation is consistent so that many transcripts are seen on the diagonal line. However, there are also a certain amount of transcripts only enriched in one of the two methods. (JPG) [file pgen.1004432.s015.jpg]

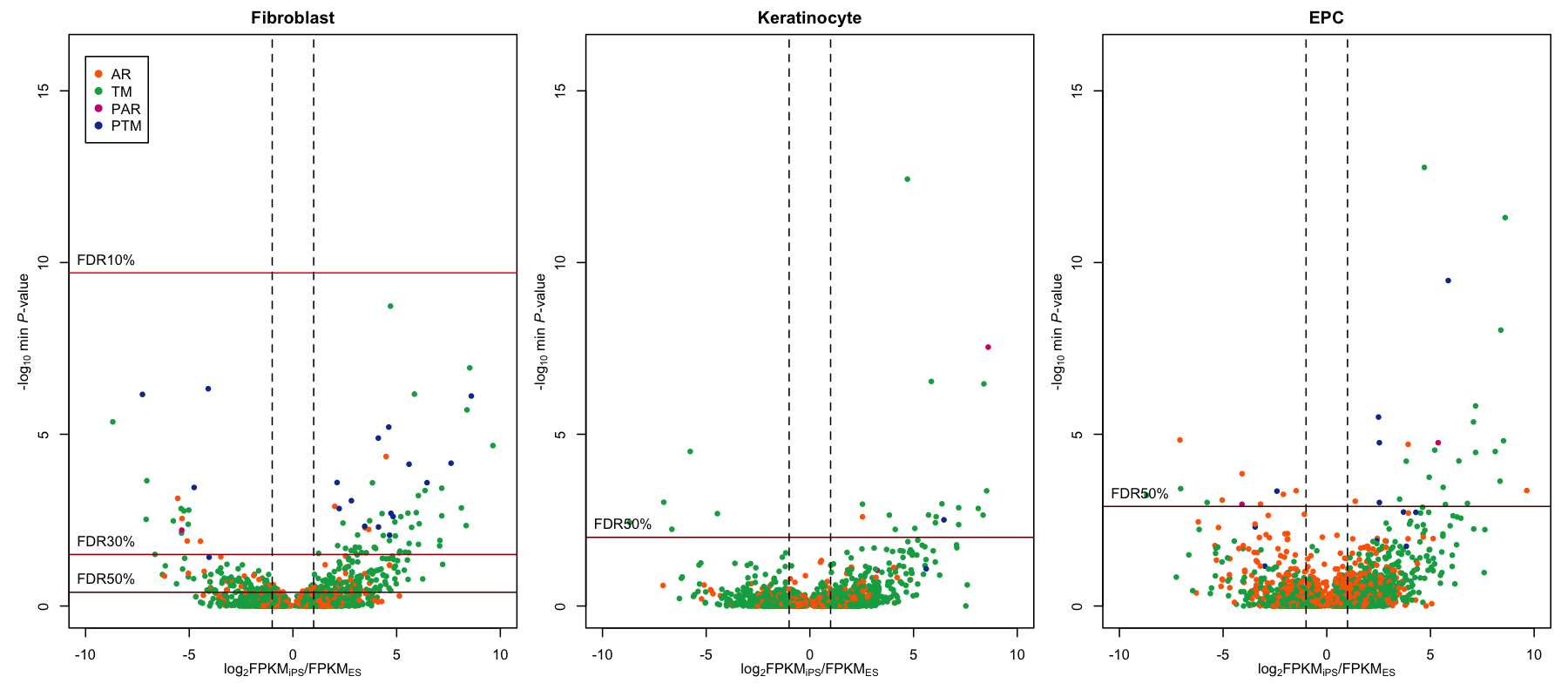

Supplement: Figure S16 — Volcano plots of differential isoform expression. Plots show log10 of minimum P-values among the four alternative hypotheses against the maximum log2 fold-change of average transcript expression levels between iPSCs and ESCs. The colours of the points indicate the differential expression categories into which a gene was classified. Dashed lines show twofold enrichment of mean expression levels between iPSCs and ESCs. The FDR threshold was calculated by the permutation scheme as in the differential expression analysis. (PNG) [file pgen.1004432.s016.png]

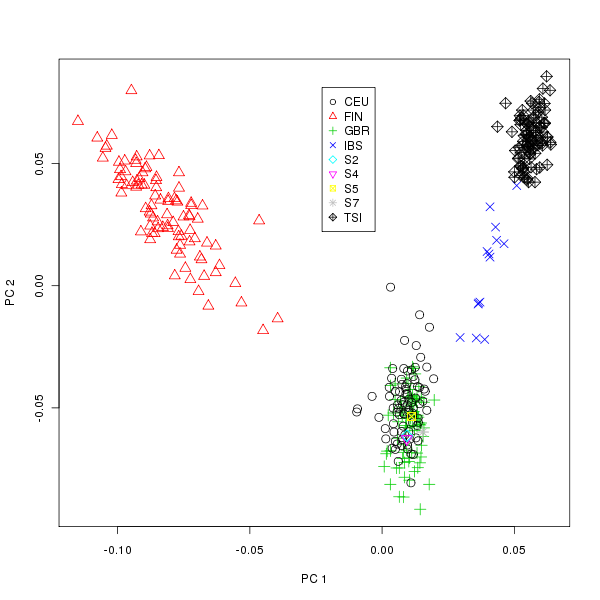

Supplement: Figure S17 — Result of population stratification for our samples (S2/S4/S5/S9) with 1000 Genomes Project data. Principal component analysis was performed using Eigenstrat [35] with genome-wide SNP genotypes of European populations obtained from 1000 Genomes Project. All four samples are clustered with GBR and CEU populations. (PNG) [file pgen.1004432.s017.png]

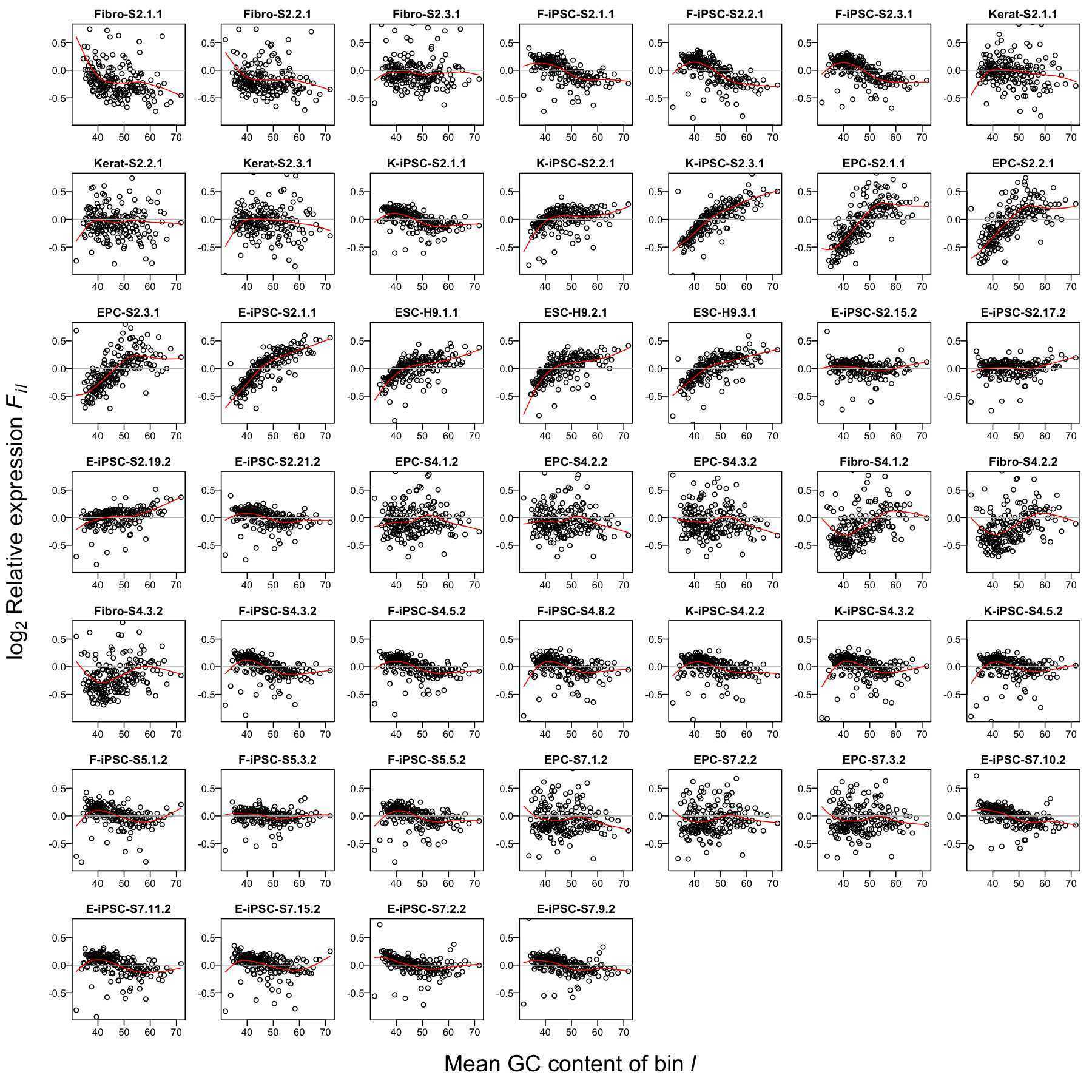

Supplement: Figure S18 — GC content influences inference of expression levels from RNA-seq. Plotted is the log2 relative enrichment, Fil, against the mean GC content of bin l for all samples. The red line shows the fitted spline function, Fil (see Text S1 for details). (JPG) [file pgen.1004432.s018.jpg]

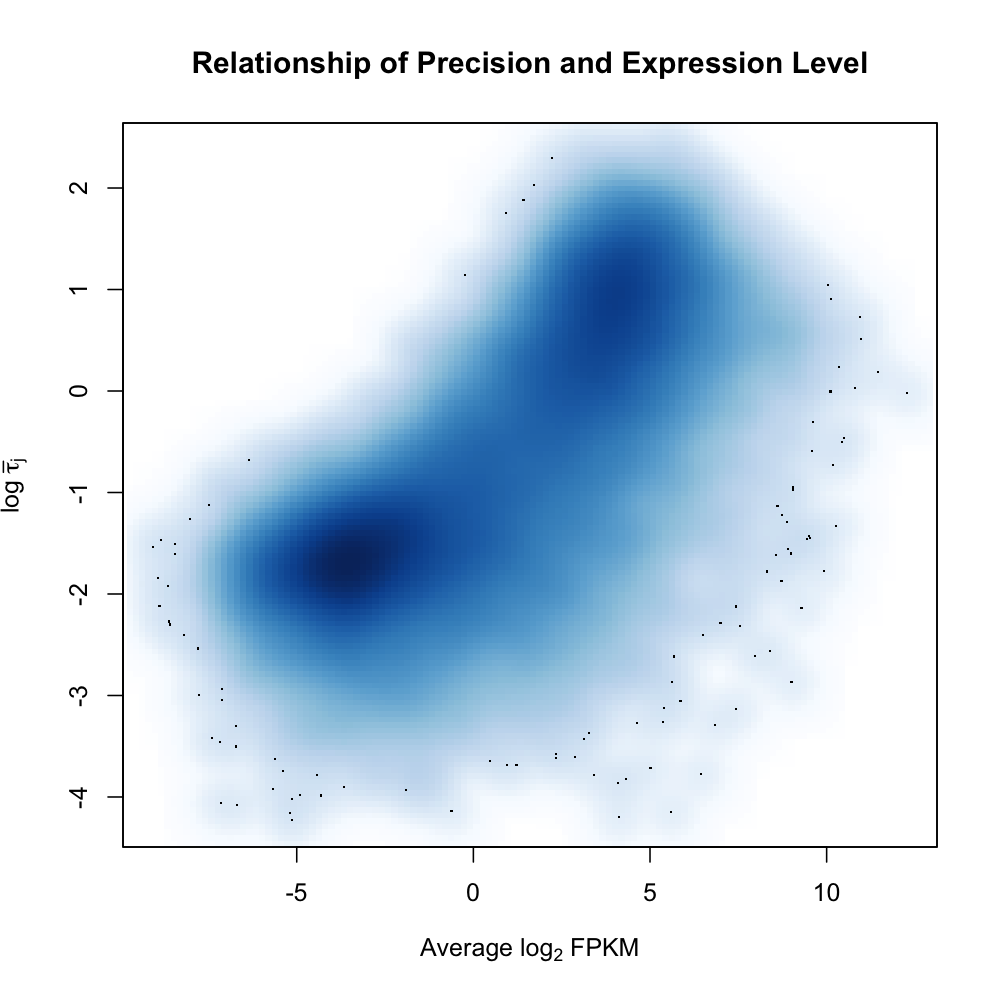

Supplement: Figure S19 — Relationship between precision and expression level. Plotted is the distribution of log τj against average of log2 normalised FPKMs y ˜j across all samples (j = 1,…, L). (PNG) [file pgen.1004432.s019.png]
