# Supplementary material for: Genetic Background Drives Transcriptional Variation in Human Induced Pluripotent Stem Cells
Source: PLoS Genet. 2014 Jun 5;10(6):e1004432. doi: 10.1371/journal.pgen.1004432 (PMC4046971; doi:10.1371/journal.pgen.1004432)
Supplement: Figure S2 — RT-PCR for Sendai viral genome and transgenes in a subset of lines. Gels show results of RT-PCR using viral primer sets as described in the CytoTune-iPS reprogramming kit (Invitrogen) in line S4SK4 (passage 3). Results are shown are for Sendai virus genome (SeV: 181 bp amplicon), Sendai-derived exogenous Oct3/4 (O: 483 bp amplicon), Sox2 (S: 451 bp am- plicon), Klf4 (K: 410 bp amplicon) and cMyc (M: 532 bp amplicon). (PDF) [file pgen.1004432.s002.pdf]

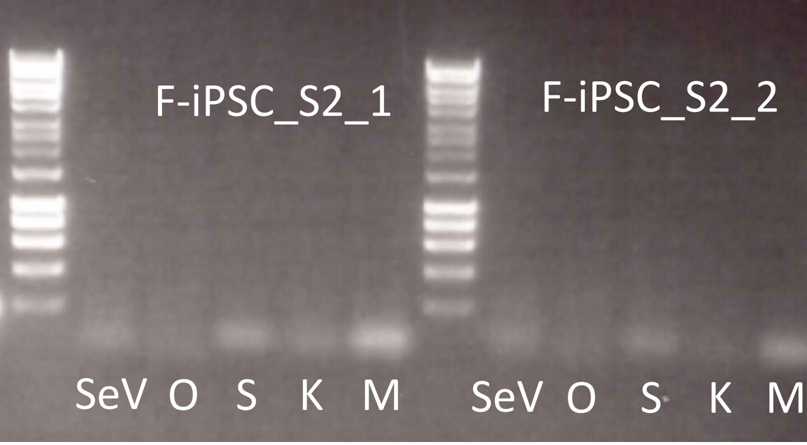
F-iPSC_S2_1
F-iPSC_S2_2
SeV O S K M
SeV O S K M
