# Supplementary material for: Genetic Background Drives Transcriptional Variation in Human Induced Pluripotent Stem Cells
Source: PLoS Genet. 2014 Jun 5;10(6):e1004432. doi: 10.1371/journal.pgen.1004432 (PMC4046971; doi:10.1371/journal.pgen.1004432)
Supplement: Figure S7 — Variance component analysis and differential expression (DE) analysis excluding highly expressed genes (upper 1%-tile). (a) Correlation heatmap without upper 1%-tile highly expressed genes (b) Result of variance component analysis without upper 1%-tile highly expressed genes. (c) P-value comparison with original DE analysis. Each panel shows scatter plot of the DE minimum P-values without upper 1%ile highly expressed genes (X-axis) against original minimum DE P-values (Y-axis) for each tissue. Gray vertical and horizontal lines show 5% FDR. (PDF) [file pgen.1004432.s007.pdf]

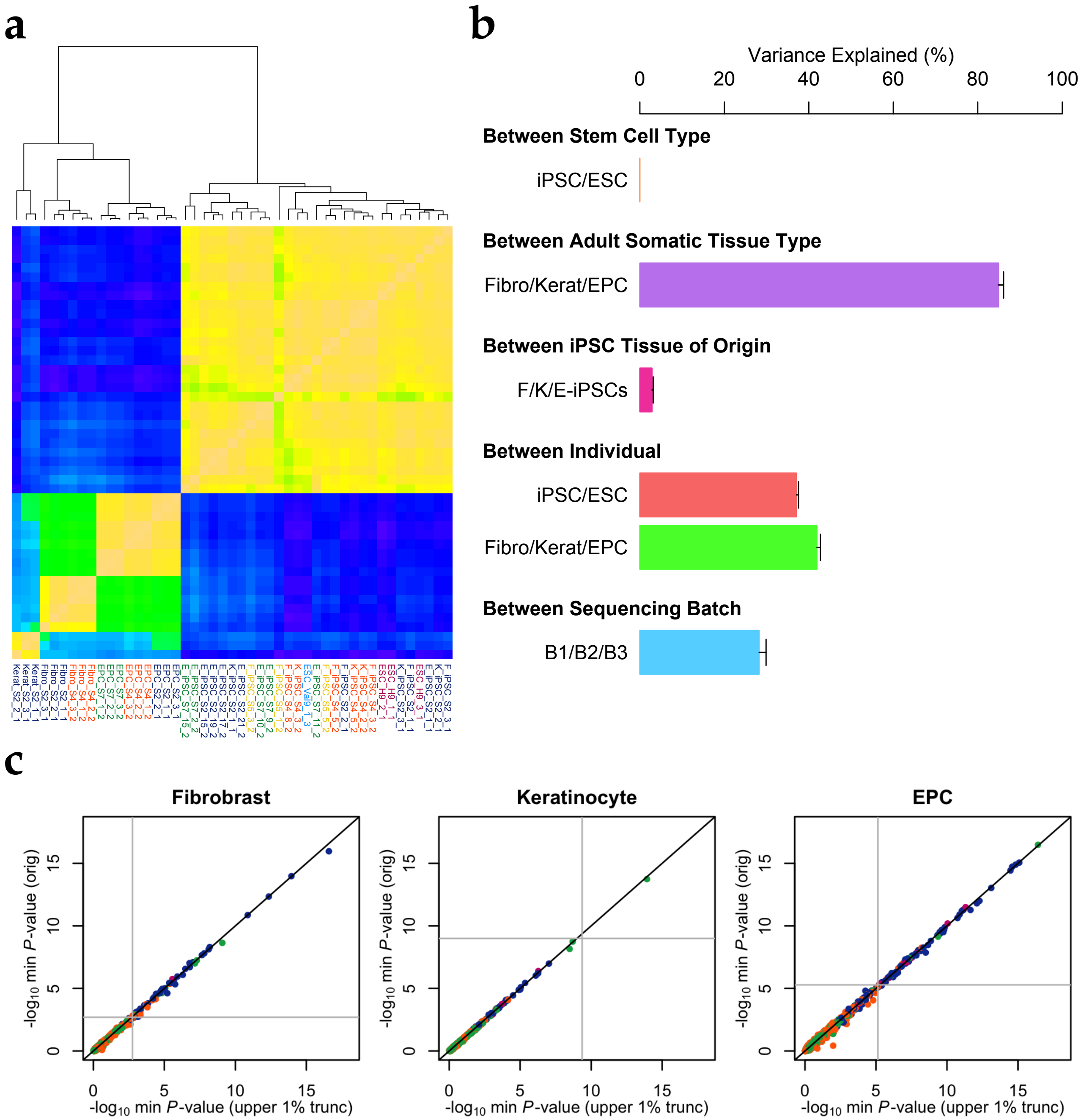
a
Kerat_S2_2_1
Kerat_S2_3_1
Kerat_S2_1_1
Fibro_S2_3_1
Fibro_S2_2_1
Fibro_S2_1_1
Fibro_S4_3_2
Fibro_S4_1_2
Fibro_S4_2_2
EPC_S7_1_2
EPC_S7_2_2
EPC_S7_3_2
EPC_S4_3_2
EPC_S4_2_2
EPC_S4_1_2
EPC_S2_2_1
EPC_S2_1_1
EPC_S2_3_1
E_iPSC_S7_15_2
E_iPSC_S7_2_2
E_iPSC_S2_15_2
E_iPSC_S2_19_2
E_iPSC_S2_17_2
K_iPSC_S2_1_1
E_iPSC_S2_21_2
F_iPSC_S5_3_2
E_iPSC_S7_10_2
E_iPSC_S7_9_2
F_iPSC_S5_1_2
F_iPSC_S4_8_2
K_iPSC_S4_3_2
ESC_Val9_1_3
E_iPSC_S7_11_2
F_iPSC_S5_5_2
F_iPSC_S4_5_2
F_iPSC_S2_2_1
K_iPSC_S4_5_2
K_iPSC_S4_2_2
F_iPSC_S4_3_2
ESC_H9_2_1
ESC_H9_1_1
K_iPSC_S2_3_1
F_iPSC_S2_1_1
ESC_H9_3_1
E_iPSC_S2_1_1
K_iPSC_S2_2_1
F_iPSC_S2_3_1
b
Variance Explained (%)
0
20
40
60
80
100
Between Stem Cell Type
iPSC/ESC
Between Adult Somatic Tissue Type
Fibro/Kerat/EPC
Between iPSC Tissue of Origin
F/K/E-iPSCs
Between Individual
iPSC/ESC
Fibro/Kerat/EPC
Between Sequencing Batch
B1/B2/B3
c
Fibrobrast
Keratinocyte
EPC
$-\log_{10}$ min P-value (orig)
$-\log_{10}$ min P-value (upper 1% trunc)
0
5
10
15
