# Supplementary material for: Genetic Background Drives Transcriptional Variation in Human Induced Pluripotent Stem Cells
Source: PLoS Genet. 2014 Jun 5;10(6):e1004432. doi: 10.1371/journal.pgen.1004432 (PMC4046971; doi:10.1371/journal.pgen.1004432)
Supplement: Figure S11 — Heatmaps of log2 FPKMs for partial transcriptional memory (PTM) genes deter- mined by the differential expression analysis. (a) Partial transcriptional memory genes in F- iPSCs, (b) K-iPSCs and (c) E-iPSCs. We note that, although patterns of expression across most lines are broadly consistent with one another, line K-iPSC-S2-1-1 forms an outlier from the other K-iPSCs (PDF) [file pgen.1004432.s011.pdf]

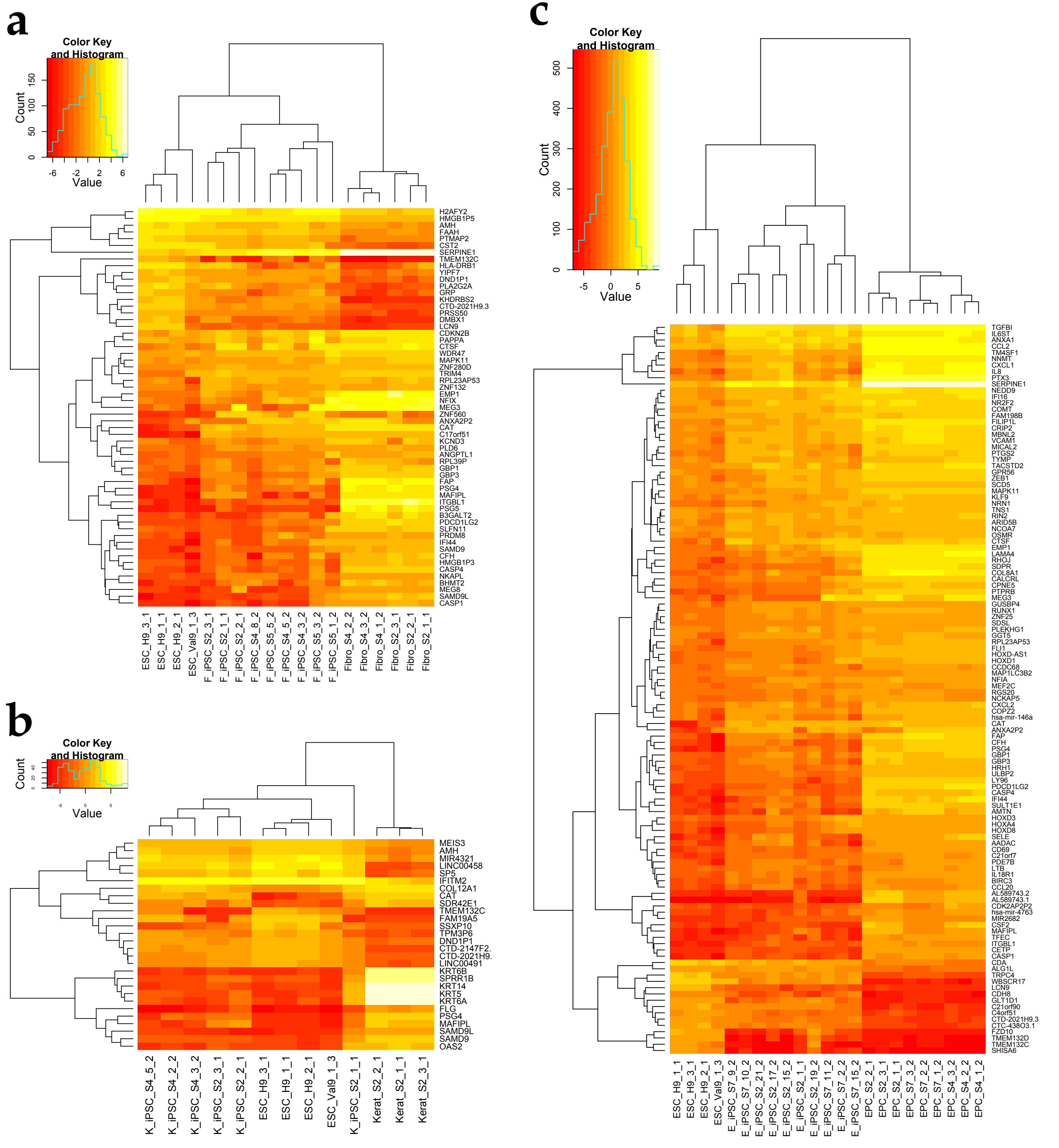
a
Color Key and Histogram
Count
Value
H2AFY2
HMGB1P5
AMH
FAAH
PTMAP2
CST2
SERPINE1
TMEM132C
HLA-DRB1
YIPF7
DND1P1
PLA2G2A
GRP
KHDRBS2
CTD-2021H9.3
PRSS50
DMBX1
LCN9
CDKN2B
PAPPA
CTSF
WDR47
MAPK11
ZNF280D
TRIM4
RPL23AP53
ZNF132
EMP1
NFIX
MEG3
ZNF560
ANXA2P2
CAT
C17orf51
KCND3
PLD6
ANGPTL1
RPL39P
GBP1
GBP3
FAP
PSG4
MAFIPL
ITGBL1
PSG5
B3GALT2
PDCD1LG2
SLFN11
PRDM8
IFI44
SAMD9
CFH
HMGB1P3
CASP4
NKAPL
BHMT2
MEG8
SAMD9L
CASP1
ESC_H9_3_1
ESC_H9_1_1
ESC_H9_2_1
ESC_Val9_1_3
F_iPSC_S2_3_1
F_iPSC_S2_1_1
F_iPSC_S2_2_1
F_iPSC_S4_8_2
F_iPSC_S5_5_2
F_iPSC_S4_5_2
F_iPSC_S4_3_2
F_iPSC_S5_3_2
F_iPSC_S5_1_2
Fibro_S4_2_2
Fibro_S4_3_2
Fibro_S4_1_2
Fibro_S2_3_1
Fibro_S2_2_1
Fibro_S2_1_1
b
Color Key and Histogram
Count
Value
MEIS3
AMH
MIR4321
LINC00458
SP5
IFITM2
COL12A1
CAT
SDR42E1
TMEM132C
FAM19A5
SSXP10
TPM3P6
DND1P1
CTD-2147F2.
CTD-2021H9.
LINC00491
KRT6B
SPRR1B
KRT14
KRT5
KRT6A
FLG
PSG4
MAFIPL
SAMD9L
SAMD9
OAS2
K_iPSC_S4_5_2
K_iPSC_S4_2_2
K_iPSC_S4_3_2
K_iPSC_S2_3_1
K_iPSC_S2_2_1
ESC_H9_3_1
ESC_H9_1_1
ESC_H9_2_1
ESC_Val9_1_3
K_iPSC_S2_1_1
Kerat_S2_2_1
Kerat_S2_1_1
Kerat_S2_3_1
c
Color Key and Histogram
Count
Value
TGFBI
IL6ST
ANXA1
CCL2
TM4SF1
NNMT
CXCL1
IL8
PTX3
SERPINE1
NEDD9
IFI16
NR2F2
COMT
FAM198B
FILIP1L
CRIP2
MBNL2
VCAM1
MICAL2
PTGS2
TYMP
TACSTD2
GPR56
ZEB1
SCD5
MAPK11
KLF9
NRN1
TNS1
RIN2
ARID5B
NCOA7
OSMR
CTSF
EMP1
LAMA4
RHOJ
SDPR
COL8A1
CALCRL
CPNE5
PTPRB
MEG3
GUSBP4
RUNX1
ZNF25
SDSL
PLEKHG1
GGT5
RPL23AP53
FLI1
HOXD-AS1
HOXD1
CCDC68
MAP1LC3B2
NFIA
MEF2C
RGS20
NCKAP5
CXCL2
COPZ2
hsa-mir-146a
CAT
ANXA2P2
FAP
CFH
PSG4
GBP1
GBP3
HRH1
ULBP2
LY96
PDCD1LG2
CASP4
IFI44
SULT1E1
AMTN
HOXD3
HOXA4
HOXD8
SELE
AADAC
CD69
C21orf7
PDE7B
LTB
IL18R1
BIRC3
CCL20
AL589743.2
AL589743.1
CDK2AP2P2
hsa-mir-4763
MIR2682
CSF2
MAFIPL
TFEC
ITGBL1
CETP
CASP1
CDA
ALG1L
TRPC4
WBSCR17
LCN9
CDH8
GLT1D1
C21orf90
C4orf51
CTD-2021H9.3
CTC-438O3.1
FZD10
TMEM132D
TMEM132C
SHISA6
ESC_H9_1_1
ESC_H9_3_1
ESC_H9_2_1
ESC_Val9_1_3
E_iPSC_S7_9_2
E_iPSC_S7_10_2
E_iPSC_S2_21_2
E_iPSC_S2_17_2
E_iPSC_S2_15_2
E_iPSC_S2_1_1
E_iPSC_S2_19_2
E_iPSC_S7_11_2
E_iPSC_S7_2_2
E_iPSC_S7_15_2
EPC_S2_2_1
EPC_S2_3_1
EPC_S2_1_1
EPC_S7_3_2
EPC_S7_2_2
EPC_S7_1_2
EPC_S4_3_2
EPC_S4_2_2
EPC_S4_1_2
