# Supplementary material for: Genetic Background Drives Transcriptional Variation in Human Induced Pluripotent Stem Cells
Source: PLoS Genet. 2014 Jun 5;10(6):e1004432. doi: 10.1371/journal.pgen.1004432 (PMC4046971; doi:10.1371/journal.pgen.1004432)
Supplement: Text S1 — Supplementary methods. (PDF) [file pgen.1004432.s020.pdf]

Contents

1 Supplementary Methods **2**
1.1 Fragment count and FPKM calculation 2
1.2 GC correction 2
1.3 Variance component analysis using a linear mixed model 2
1.3.1 Scaling of gene expression levels 4
1.3.2 Parameter estimation 5
1.3.3 Heteroscedastic model 6
1.3.4 Estimation of Primary eQTL SNP variation 7
1.4 Differential Expression Analysis 8
1.4.1 Gene selection 9
1.4.2 *P*-value method using DESeq 9
1.4.3 Hierarchical model 9
1.4.4 Gene ontology analysis 11
1.4.5 Transposable element transcription 11
1.5 Allele-specific Expression Analysis 12
1.5.1 Comparison with eQTL analysis in Lappalainen *et al.* 13

1 Supplementary Methods

1.1 Fragment count and FPKM calculation

For each gene in the gene annotation, we counted the number of sequenced fragments of which one or other sequenced end lay within an annotated ENSEMBL gene exon. Let Y_{ij} be the fragment count of the gene j ($j = 1, \ldots, L$) for a sample i ($i = 1, \ldots, N$). We had a total $L = 55,804$ genes from our gene annotation and a sample size of $N = 46$. We calculated $\log_2$ FPKM (fragments per kilobase of exon per million fragments mapped), y_{ij}, for sample i at gene j as follows:

$$y_{ij} = \log_2 \left(\frac{Y_{ij} + 1}{l_j Y_i} \right),$$

where l_j is the mean spliced transcript length in kilobase of gene j calculated from the gene annotations and $Y_i = \sum_{j=1}^{L} Y_{ij}/10^6$ is the total fragment count in megabase for the sample i.

1.2 GC correction

We corrected for varying amplification efficiency of different GC contents using the method described in [2]. We first calculated mean GC content for each gene, which is mean G/C base counts over mean cDNA length from all possible transcripts of a gene in our annotation. Then we assigned all genes to 200 approximately equally sized bins $\{\mathcal{B}_1, \ldots, \mathcal{B}_{200}\}$ based on the GC content. Let $S_{il} = \sum_{j \in \mathcal{B}_l} Y_{ij}$ be the number of fragments in bin l from sample i. For each bin, for each sample, we calculated the $\log_2$ relative enrichment, F_{il}, of fragments in each GC bin, such that

$$F_{il} = \log_2 \left(\frac{S_{il}/S_{\cdot l}}{S_{i\cdot}/S_{\cdot\cdot}} \right),$$

where $S_{\cdot l} = \sum_i S_{il}$, $S_{i\cdot} = \sum_l S_{il}$ and $S_{\cdot\cdot} = \sum_{i,l} S_{il}$. For each sample, we fitted a smoothing spline to the plot of F_{il} against the mean GC content for the bin. We used the `R` function `smooth.spline` with a smoothing parameter of 1. The result showed significant GC effect on each sample (Supplementary Figure 18).

Letting $\hat{F}_{il}$ be the predicted value of the smoothing spline for bin l in sample i, we set $c_{ij} = \hat{F}_{il}$, where c_{ij} is the predicted $\log_2$ over/under-representation of fragment count of gene $j \in \mathcal{B}_l$ in sample i. Then the normalised FPKM was obtained by

$$\tilde{y}_{ij} = y_{ij} - c_{ij}.$$

For simplicity, we calculated FPKMs based on genes instead of exons.

1.3 Variance component analysis using a linear mixed model

Although hierarchical clustering illustrated major sources of transcriptional variations between adult and stem cells or between tissues in adult cells, more subtle sources of variation, such as

tissue of origin variation in iPSCs or ES/iPS variation, were difficult to discern. To provide quantitative information on the relative importance of different sources of variation we employed the following variance components analysis using a linear mixed model. We assumed that the normalised $\log_2$ FPKMs for gene j, $\tilde{y}_j = (\tilde{y}_{1j}, \ldots, \tilde{y}_{Nj})^\top$, can be modelled as a linear combination of fixed and normally distributed random effects, such that

$$\tilde{y}_j = Z_1 b_{1j} + Z_2 b_{2j} + Z_3 b_{3j} + Z_4 b_{4j} + Z_5 b_{5j} + \varepsilon_j \qquad (j = 1, \ldots, L) \tag{1}$$

where

$b_{1j} \overset{i.i.d.}{\sim} \mathcal{N}(\beta_1, D_1/\tau_j)$: Mixed intercept (Adult cell/Stem cell)

$b_{2j} \overset{i.i.d.}{\sim} \mathcal{N}(\beta_2, D_2/\tau_j)$: Between stem cell types (iPSC/ESC)

$b_{3j} \overset{i.i.d.}{\sim} \mathcal{N}(\beta_3, D_3/\tau_j)$: Between tissues for adult cells (F/K/E)/tissue of origin for iPSCs (iF/iK/iE)

$b_{4j} \overset{i.i.d.}{\sim} \mathcal{N}(\beta_4, D_4/\tau_j)$: Between individual for adult cells (S2/S4/S7) and iPSCs (S2/S4/S5/S7/H9)

$b_{5j} \overset{i.i.d.}{\sim} \mathcal{N}(\beta_5, D_5/\tau_j)$: Between sequencing batches (B1/B2)

$\varepsilon_j \overset{i.i.d.}{\sim} \mathcal{N}(0, \Sigma/\tau_j)$: Biological and Technical Error

with variance-covariance matrices

$$\begin{aligned}
D_1 &= \begin{pmatrix} \delta_{11}^2 & \delta_{12}^2 \\ \delta_{12}^2 & \delta_{13}^2 \end{pmatrix}, \\
D_2 &= \delta_2^2 I_2, \\
D_3 &= \begin{pmatrix} \delta_{31}^2 I_3 & 0 \\ 0 & \delta_{32}^2 I_3 \end{pmatrix}, \\
D_4 &= \begin{pmatrix} \delta_{41}^2 I_3 & 0 \\ 0 & \delta_{42}^2 I_5 \end{pmatrix}, \\
D_5 &= \delta_5^2 I_2, \\
\Sigma &= \sigma^2 I_N,
\end{aligned}$$

where $Z_1, \ldots, Z_5$ are design matrices of 0/1 values, in each of which ith row specifies the category that the sample i belongs to, and I_d indicates the d-dimensional identity matrix.

The first term in the model was an intercept, b_{1j}. Because the primary goal of this part of our analysis was to quantify variation rather than estimate mean expression levels, we estimated the parameters of the distribution from which the means were drawn, rather than the means themselves. The intercept term captures three properties of the distribution of gene expression levels, subdivided into two groups for adult and stem cells: the grand means of gene expression across all genes, corresponding to the two elements of the vector β_1, the variances of gene expression across all genes corresponding to the first and second diagonal elements of the

two-dimensional variance matrix D_1, δ^2_{11} and δ^2_{12} and the covariances in expression levels corresponding to the off-diagonal elements of the variance matrix D_1, δ^2_{12}. The second term, b_{2j}, was specific to only stem cells and captured variation between iPS and ES cells (variance parameter δ^2_2) The third term, b_{3j}, captured variation between the different adult somatic cells (variance parameter δ^2_{31}) and between different somatic tissues of origin for iPSCs (variance parameter δ^2_{31}). The fourth term, b_{4j} captured the variation between individuals. We were specifically interested in whether between-individual variation differed between adult and iPS cells and so we assigned different variance parameters for each, δ^2_{41} and δ^2_{42} for adult and stem cells, respectively. The final term, b_{5j}, captured variation between different sequencing batches. We assumed sequencing batch was independent of cell or tissue type, and so we introduced a common variance parameter δ^2_5 for this term. The fixed effect parameters $\beta_2, \ldots, \beta_5$ captured grand means of expression levels under specific conditions (cell type, tissue type, etc.). However, because of our unbalanced study design, the fixed effect parameters are not uniquely determined and their biological interpretability is therefore limited. We note that estimates of the variance parameters are still uniquely determined in an unbalanced sampling design.

	Computation of variance Explained (intraclass correlation)
**Between Stem Cell Type**	
iPSC/ESC	$\frac{\delta^2_2}{\delta^2_2 + \sigma^2}$
**Between Adult Somatic Tissue Type**	
Fibro/Kerat/EPC	$\frac{\delta^2_{31}}{\delta^2_{31} + \sigma^2}$
**Between iPSC Tissue of Origin**	
F/K/E-iPSCs	$\frac{\delta^2_{32}}{\delta^2_{32} + \sigma^2}$
**Between Individual**	
iPSC/ESC	$\frac{\delta^2_{41}}{\delta^2_{41} + \sigma^2}$
Fibro/Kerat/EPC	$\frac{\delta^2_{42}}{\delta^2_{42} + \sigma^2}$
**Between Sequencing Batch**	
B1/B2/B3	$\frac{\delta^2_5}{\delta^2_5 + \sigma^2}$

1.3.1 Scaling of gene expression levels

A standard feature of gene expression data is a greater variance in expression in lowly expressed genes (see *e.g.*, [8]). To capture this aspect of our data, all variance parameters $\delta =$

$(\delta_{11}, \delta_{12}, \delta_{13}, \delta_{21}, \delta_{22}, \delta_{31}, \delta_{32}, \delta_{41}, \delta_{42}, \delta_5, \sigma)$ were scaled by precision

$$\tau_j \stackrel{i.i.d.}{\sim} \mathcal{G}(\nu/2, \nu/2)$$

for each gene j, which is identically and independently Gamma distributed.

In our data, the posterior mean of τ_j given by (2) clearly showed that the higher the expression level is the higher the precision is (Supplementary Figure 19). Technically, the assumption that τ follows a Gamma distribution provided two advantages. First, this limited the range of τ_j in $(0, \infty)$ thereby avoiding parameter uncertainty. Second, the Gamma distribution is a conjugate prior distribution for $\tilde{y}_j$ given τ_j, such that

$$\tilde{y}_j|\tau_j \sim \mathcal{N}(Z\beta, V/\tau_j),$$

where $V = ZDZ^\top + \Sigma$ with the block diagonal matrix $D = \text{diag}(D_1, \ldots, D_5)$, the compound fixed effect vector $\beta^\top = (\beta_1^\top, \ldots, \beta_5^\top)$ and the combined design matrix $Z = (Z_1, \ldots, Z_5)$, so that the marginal distribution of $\tilde{y}_j$ is the multivariate student t distribution

$$\tilde{y}_j \sim \mathcal{T}(Z\beta, V, \nu)$$

with mean $Z\beta$, variance V and the ν degrees of freedom. The multivariate student t distribution is convenient for parameter estimation using an EM algorithm.

1.3.2 Parameter estimation

We used a standard EM algorithm [10] to estimate all parameters $\theta = \{\beta, \delta, \nu\}$, where τ_j $(j = 1, \ldots, L)$ are thought to be unobserved variables. Because the Gamma distribution on τ_j is a conjugate prior for $\tilde{y}_j$ given τ_j, the posterior distribution of τ_j given $\tilde{y}_j$ is also a Gamma distribution such that

$$\tau_j|\tilde{y}_j \sim \mathcal{G}\left(\frac{N+\nu}{2}, \frac{(\tilde{y}_j - Z\beta)^\top V^{-1}(\tilde{y}_j - Z\beta) + \nu}{2}\right).$$

Therefore the posterior means

$$\mathbb{E}[\tau_j|\tilde{y}_j] = \overline{\tau}_j = \frac{N+\nu}{(\tilde{y}_j - Z\beta)^\top V^{-1}(\tilde{y}_j - Z\beta) + \nu} \quad (2)$$

$$\mathbb{E}[\log \tau_j|\tilde{y}_j] = \overline{\log \tau}_j = \psi[(N+\nu)/2] - \log[\{(\tilde{y}_j - Z\beta)^\top V^{-1}(\tilde{y}_j - Z\beta) + \nu\}/2]$$

can be obtained without any numerical integration in each E-step, where $\psi[\cdot]$ is the digamma function.

In the M-step, we alternatively maximised a series of Q-functions using a Newton-Raphson method with the posterior means of τ_j and $\log \tau_j$, such that

$$Q(\beta|\hat{\theta}) = -\sum_{j=1}^{L} \frac{\overline{\tau}_j}{2}(\tilde{y}_j - Z\beta)^\top V^{-1}(\tilde{y}_j - Z\beta),$$

$$Q(\delta|\hat{\theta}) = -\sum_{j=1}^{L} \frac{\overline{\tau}_j}{2}(\tilde{y}_j - Z\beta)^\top V^{-1}(\tilde{y}_j - Z\beta) - \frac{L}{2}\log|V|,$$

$$Q(\nu|\hat{\theta}) = \sum_{j=1}^{L} \frac{\nu}{2}\overline{\log \tau_j} - \sum_{j=1}^{L} \frac{\overline{\tau}_j}{2}\nu + L\frac{\nu}{2}\log\frac{\nu}{2} - L\log\Gamma(\nu/2),$$

with respect to the parameters β, δ and ν. Although the mean $Z\hat{\beta}$ is not of interest here, it is required to estimate δ and ν. Because Z is essentially degenerate, β is not uniquely determined and, therefore, to maximise the likelihood function, we use a generalised inverse matrix $A^+ = A^+AA^+$ to obtain the maximum likelihood estimator

$$\hat{\beta} = (Z^\top V^{-1} Z)^+ Z^\top V^{-1} \eta$$

where

$$\eta = \frac{\sum_{j=1}^{L} \overline{\tau}_j \tilde{y}_j}{\sum_{j=1}^{L} \overline{\tau}_j}.$$

1.3.3 Heteroscedastic model

Our initial model assumed that the residual error has the same variance for all factors in the model (homoscedasticity). We next extended this model to allow the residuals in different cell types to take different variances, known as heteroscedasticity.We incorporated a variety of different error parameters on each ε_{ij} according to the cell/tissue type of the sample i, including

$$\mathrm{Var}(\varepsilon_{ij}) = \begin{cases} \sigma^2_{\mathrm{adult}}/\tau_j & \text{Sample } i \text{ is an adult cell} \\ \sigma^2_{\mathrm{iPS}}/\tau_j & \text{iPS cell} \\ \sigma^2_{\mathrm{ES}}/\tau_j & \text{ES cell} \end{cases}$$

or

$$\mathrm{Var}(\varepsilon_{ij}) = \begin{cases} \sigma^2_{\mathrm{F}}/\tau_j & \text{Sample } i \text{ is fibroblast} \\ \sigma^2_{\mathrm{K}}/\tau_j & \text{keratinocyte} \\ \sigma^2_{\mathrm{E}}/\tau_j & \text{endothelial precursor cell (EPC)} \\ \sigma^2_{\mathrm{iF}}/\tau_j & \text{iPSC derived from fibroblast} \\ \sigma^2_{\mathrm{iK}}/\tau_j & \text{iPSC from keratinocyte} \\ \sigma^2_{\mathrm{iE}}/\tau_j & \text{iPSC from EPC} \\ \sigma^2_{\mathrm{ES}}/\tau_j & \text{ESC} \end{cases}$$

as in Fig. 1d in the main text.

1.3.4 Estimation of Primary eQTL SNP variation

To further dissect the individual variance into genetic and non-genetic variances, we introduced an additional covariate in (1), which is the primary eQTL SNP found in gEUVADIS project [15]. Because the SNP genotype is only available for iPS lines but not for ES lines in our study, we modelled the FPKM for 25 iPS lines, such that

$$\tilde{y}_j = b_{1j}1 + Z_3 b_{3j} + Z_4 b_{4j} + Z_5 b_{5j} + b_{6j}\tilde{g}_j + \varepsilon_j,$$

where b_{1j} is a scalar mixed intercept drawn from $\mathcal{N}(\alpha_1, \delta_1^2/\tau_j)$ and 1 is a vector of all 1s. Following $b_{3j}, \ldots, b_{5j}$ and $Z_3, \ldots, Z_5$ are the mixed effects and the design matrices defined as before. We don't have the component b_{2j} because we have no ES lines in the model. The vector $\tilde{g}_j$ is the normalised SNP genotype at the primary eQTL for the gene j. We first estimated the population allele frequency $\hat{\pi}_j$ from the SNP genotypes of CEU and GBR populations in the 1000 Genomes Project and then normalised the raw SNP genotype g_j of ours by

$$\tilde{g}_j = \frac{g_j - (2\hat{\pi}_j)1}{\sqrt{2\hat{\pi}_j(1-\hat{\pi}_j)}}$$

so that

$$\hat{K} = \frac{1}{2L}\sum_{j=1}^{L} \tilde{g}_j \tilde{g}_j^\top$$

becomes an unbiased, positive semi-definite estimator for the kinship matrix [14].

There is a problem when we compare the variance explained by the component with others, because the variance is essentially different for each individual i at gene j;

$$\mathrm{Var}(b_{j6}\tilde{g}_{ij}|\tau_j) = \tilde{g}_{ij}^2 \hat{\sigma}_g^2 \tau_j^{-1}. \tag{3}$$

One solution would be to calculate the overall transcriptional variation given the set of eQTL SNPs. By integrating out τ_j from (3), such that

$$\mathrm{Var}(b_{j6}\tilde{g}_{ij}) = \int \tilde{g}_{ij}^2 \hat{\sigma}_g^2 \tau_j^{-1} p(\tau_j) d\tau_j = \tilde{g}_{ij}^2 \hat{\sigma}_g^2 \frac{\hat{\nu}}{\hat{\nu}-2},$$

we have the averaged variance across all genes

$$\begin{aligned}\frac{1}{L}\sum_{j=1}^{L}\mathrm{Var}(b_{j6}\tilde{g}_{ij}) &= \left(\frac{1}{L}\sum_{j=1}^{L}\tilde{g}_{ij}^2\right)\hat{\sigma}_g^2\frac{\hat{\nu}}{\hat{\nu}-2} \\ &= 2\hat{K}_{ii}\hat{\sigma}_g^2\frac{\hat{\nu}}{\hat{\nu}-2} \\ &\approx \hat{\sigma}_g^2\frac{\hat{\nu}}{\hat{\nu}-2},\end{aligned}$$

where the diagonal element $\hat{K}_{ii} \to 1/2$ as $L \to \infty$ for an outbred individual. The averaged variance is compatible with the averaged residual variance

$$\frac{1}{L}\sum_{j=1}^{L} \mathrm{Var}(\varepsilon_j) = \hat{\sigma}^2 \frac{\hat{\nu}}{\hat{\nu}-2}.$$

Therefore the overall variance explained for the set of eQTL SNPs is given by $\hat{\sigma}_g^2/(\hat{\sigma}_g^2 + \hat{\sigma}^2)$.

1.4 Differential Expression Analysis

Although our variance components analysis suggested relatively small effects of tissue of origin, this could mask subtle effects at important individual genes. We next sought to identify genes whose expression in iPSCs more closely resembled their somatic progenitors or which appeared to be significantly diverged from both ESCs and somatic cells. We performed differential expression analysis using two different approaches; one is using DESeq method [8] and a novel hierarchical model similar to that introduced in [9]. For both these methods, we analysed different tissues (skin fibroblast, keratinocyte and EPC) independently.

Our analysis compared mean expression levels of all cell types (adult cell, iPSC and ESC) simultaneously. We modelled the fragment count Y_{ij} for sample i at a gene j using a negative binomial distribution, such that

$$Y_{ij} \sim \mathcal{NB}(\lambda_{ij} K_{ij}, \theta_j)$$

with the cell type specific means

$$\lambda_{ij} = \begin{cases} \lambda_j^{\mathrm{A}} & \text{Sample } i \text{ is an adult cell} \\ \lambda_j^{\mathrm{I}} & \text{iPSC} \\ \lambda_j^{\mathrm{E}} & \text{ESC} \end{cases}$$

and the offset $K_{ij} = 2^{c_{ij}} Y_i$ correcting GC content and total fragment count bias. Note that the over-dispersion parameter θ_j was treated in different way for the two approaches as described in subsequent sections. Then we introduced the null hypothesis where all cell types share the same mean expression level:

$$H_0: \quad \lambda_j^{\mathrm{A}} = \lambda_j^{\mathrm{I}} = \lambda_j^{\mathrm{E}} \qquad \text{(Invariant Expression)}$$

compared with the following four alternative hypotheses:

$$\begin{array}{lll} H_1: & \lambda_j^{\mathrm{A}} \neq \lambda_j^{\mathrm{I}} = \lambda_j^{\mathrm{E}} & \text{(Correct Reprogramming)} \\ H_2: & \lambda_j^{\mathrm{A}} = \lambda_j^{\mathrm{E}} \neq \lambda_j^{\mathrm{I}} & \text{(Aberrant Reprogramming)} \\ H_3: & \lambda_j^{\mathrm{A}} = \lambda_j^{\mathrm{I}} \neq \lambda_j^{\mathrm{E}} & \text{(Transcriptional Memory)} \\ H_4: & \lambda_j^{\mathrm{A}} \neq \lambda_j^{\mathrm{I}} \neq \lambda_j^{\mathrm{E}} \neq \lambda_j^{\mathrm{A}} & \text{(Complex)} \end{array} \qquad (4)$$

where the superscript "A", "I" or "E" stands for adult cell, iPS cell or ES cell, respectively.

1.4.1 Gene selection

We filtered out low or unexpressed genes in advance by only including those genes with mean FPKM>1 in at least one cell type (A, I or E). We also filtered out genes whose biotypes defined by Ensembl are snRNA, snoRNA, misc_RNA, miRNA and rRNA, because, those expression levels cannot be correctly captured by our RNA extraction protocol. Finally, because read alignments appeared to be unreliable in many known pseudogenes, these were also removed from our annotation.

1.4.2 *P*-value method using DESeq

To identify individual genes of interest, we used gene-by-gene *P*-value tests. We employed DESeq [8] to estimate the over-dispersion parameter θ_j across all genes under the null hypothesis where all cell types share the same mean expression level. We performed hypothesis testing for each of the four alternative hypotheses in (4) and selected the most significant hypothesis that gives the minimum *P*-value. We used `nbinomGLMTest` implemented in the DESeq package to obtain the *P*-values.

Because we selected the minimum *P*-value from four alternative hypotheses, our *P*-value distribution under the null was slightly skewed towards 0. To correct for this, we estimated an empirical false discovery rate (FDR) from one million permuted data sets, where sample labels were permuted with respect to the expression data, the same differential expression tests were performed and the minimum p-value for the four alternative hypotheses was selected. The genome-wide p-value thresholds corresponding to a 5% FDRs were then estimated separately for each tissue of origin from the empirical distributions as: Fibroblasts/F-iPSCs – $p<0.015$; Keratinocyte/K-iPSCs – $p<0.009$;EPCs/E-iPSCs – $p<0.004$).

1.4.3 Hierarchical model

Although the *P*-value method gave us a rough estimate of the proportion of each alternative hypothesis that different gene will fall into, estimating these proportions is based on an arbitrary FDR threshold, such as 5%. Therefore, to estimate the proportions of genes falling into either the null or alternative hypotheses across all genes, we employed the following hierarchical model similar to that proposed in [9].

We first introduced probability Π_0 for the invariant expression (null hypothesis) and $\Pi_1 = 1 - \Pi_0$ for differential expression (alternative hypotheses). The probability of observing fragment counts for all samples at gene j, $Y_j = (Y_{1j}, \ldots, Y_{Nj})$, was defined as

$$p(Y_j) = \Pi_0 P_j^0 + \Pi_1 P_j^1,$$

where P_j^0 denotes the probability of the fragment counts given that there is no expression difference among the three cell types and P_j^1 denotes the probability of the fragment counts given

that the gene is differentially expressed between the three cell types. Given that the gene j is differentially expressed, the probability of the observed fragment counts can be given by

$$P_j^1 = \sum_{k=1}^{4} \pi_k P_{jk}^1,$$

where P_{jk}^1 is the probability of the fragment counts observed under the alternative hypothesis k and π_k is the prior probability that a gene belongs the alternative hypothesis k such that $\sum_{k=1}^{4} \pi_k = 1$. Overall, the likelihood was given by

$$L(\Pi, \pi) = \prod_{j=1}^{L} p(Y_j)$$

and the mixture parameters $\Pi = \{\Pi_0, \Pi_1\}$ and $\pi = \{\pi_k\}_{k=1}^4$ can be estimated by using a standard EM algorithm [10]. Note that π_k in our model does not depend on j which suggests that the prior distribution is essentially non-informative.

The probability P_j^0 and $\{P_{jk}^1\}_{k=1}^4$ were assumed to be given by the following negative binomial distribution

$$Y_{ij}|\beta_j, \theta_j \sim \mathcal{NB}(\lambda_{ij} K_{ij}, \theta_j)$$

with mean parameter $\log \lambda_{ij} = x_i^\top \beta_j$, where effect size β_j was defined according to the null and alternative hypotheses, such as

$$\beta_j = \begin{cases} \beta_j^0 & \text{Invariant Expression} \\ (\beta_j^{\mathrm{A}}, \beta_j^{\mathrm{I/E}})^\top & \text{Correct Reprogramming} \\ (\beta_j^{\mathrm{I}}, \beta_j^{\mathrm{A/E}})^\top & \text{Aberrant Reprogramming} \\ (\beta_j^{\mathrm{E}}, \beta_j^{\mathrm{A/I}})^\top & \text{Transcriptional Memory} \\ (\beta_j^{\mathrm{A}}, \beta_j^{\mathrm{I}}, \beta_j^{\mathrm{E}})^\top & \text{Complex} \end{cases}$$

with a compatible design vector x_i indicating the cell type for sample i ($x_i = 1$ for the invariant expression), where the superscript "A", "I" or "E" stands for adult cell, iPS cell or ES cell, respectively and the superscripts "I/E", "A/E" or "A/I" denotes shared mean for two different cell types (*i.e.*, I/E stands for $\lambda_j^{\mathrm{I}} = \lambda_j^{\mathrm{E}}$). This definition to that in (4), the mean expression level λ_j^* was reparametrized in the logarithmic scale (*e.g.*, $\log \lambda_j^{\mathrm{A}} = \log \lambda_j^{\mathrm{I}} = \beta_j^{\mathrm{A/I}}$ and $\log \lambda_j^{\mathrm{E}} = \beta_j^{\mathrm{E}}$ for a transcriptional memory gene) so as to introduce prior distributions on β_j as well as θ_j;

$$\beta_j|\theta_j \sim \mathcal{N}(0, \Sigma/\theta_j),$$
$$\theta_j \sim \mathcal{G}(\kappa/2, \delta/2),$$

where $\mathcal{N}(\mu, \Sigma)$ denotes a normal distribution with mean μ and variance-covariance matrix Σ and $\mathcal{G}(\alpha, \beta)$ denotes a gamma distribution with shape parameter α and rate parameter β. The joint probability was then given by

$$p(Y_j, \beta_j, \theta_j) = p(\theta_j) p(\beta_j|\theta_j) \prod_i p(Y_{ij}|\beta_j, \theta_j). \tag{5}$$

In order to calculate P_j^0 and $\{P_{jk}^1\}_{k=1}^4$, we needed to integrate out β_j and θ_j from the equation. There was no closed form for the negative binomial distribution unlike the normal gamma mixture in [9]. Therefore we used the Laplace approximation such that

$$\begin{aligned}
p(Y_j|\Sigma,\kappa,\delta) &= \int p(\theta_j)p(\beta_j|\theta_j)\prod_i p(Y_{ij}|\beta_j,\theta_j)d\beta_j d\theta_j \\
&\approx \int p(\theta_j)p(\hat{\beta}_j|\theta_j)|\mathcal{I}_{\hat{\beta}_j}(\theta_j)|^{-1/2}\prod_i p(Y_{ij}|\hat{\beta}_j,\theta_j)d\theta_j \\
&\approx p(\hat{\theta}_j)p(\hat{\beta}_j|\hat{\theta}_j)|\mathcal{I}_{\hat{\beta}_j}(\hat{\theta}_j)|^{-1/2}|\mathcal{I}_{\hat{\theta}_j}|^{-1/2}\prod_i p(Y_{ij}|\hat{\beta}_j,\hat{\theta}_j),
\end{aligned}$$

where

$$\begin{aligned}
\mathcal{I}_{\beta_j}(\theta_j) &= \mathbb{E}_{Y_j}\left[-\frac{\partial^2}{\partial\beta_j\partial\beta_j^\top}\log p(Y_j,\beta_j,\theta_j)\right] \\
\mathcal{I}_{\theta_j} &= \mathbb{E}_{Y_j}\left[-\frac{\partial^2}{\partial\theta_j^2}\left\{\log p(Y_j,\hat{\beta}_j,\theta_j) - \frac{1}{2}\log|\mathcal{I}_{\hat{\beta}_j}(\theta_j)|\right\}\right]
\end{aligned}$$

Here the maximum likelihood estimator of β_j and the maximum modified profile likelihood estimator of θ_j such that

$$\begin{aligned}
\hat{\beta}_j &= \underset{\beta_j}{\operatorname{argmax}}\ \log p(Y_j,\beta_j,\theta_j), \\
\hat{\theta}_j &= \underset{\theta_j}{\operatorname{argmax}}\ \log p(Y_j,\hat{\beta}_j,\theta_j) - \frac{1}{2}\log|\mathcal{I}_{\hat{\beta}_j}(\theta_j)|,
\end{aligned}$$

were obtained by using a quasi-Newton method.

The hyperparameters Σ, κ and δ were not integrated out from the joint probability in (5), instead, we assigned $\Sigma = 10^3 I$ and $\kappa = \delta = 10^{-3}$ suggesting the prior distributions were almost non-informative compared with the range of $\hat{\beta}_j$ and $\hat{\theta}_j$ across all genes (data not shown).

1.4.4 Gene ontology analysis

Genes that were significantly differentially expressed (either transcriptional memory or aberrant reprogramming) at a 5% FDR, and showed a more than a 2-fold difference in expression between ESCs and IPSCs were selected for Gene Ontology analysis. All expressed genes in each tissue, their derived iPSCs or the ESCs were used as a background. For example, in F-iPSCs, any gene that was defined as expressed in either fibroblasts, F-iPSCs or ESCs was used as the background. All GO analysis was performed using the topGO R package [12]

1.4.5 Transposable element transcription

We downloaded annotated LINE and LTR repetitive elements as defined by the UCSC genome browser. We removed all elements that overlapped an annotated transcribed region as defined

in Ensembl GRCh37 assemby 69. Using these criteria we identified 533,254 and 305,339 LINE and LTR elements, respectively, in the human genome. We further removed any elements whose average mapability for 75bp fragments was less than 1, as defined in the wgEncode-CrgMapabilityAlign75mer annotation available from UCSC and counted the total number of fragments that overlapped each annotated repetitive element in our data.

1.5 Allele-specific Expression Analysis

We next focused on the effects of varying genetic background in our iPSCs. Our initial variance component analysis suggested that differences between individuals had a more significant impact on genome-wide gene expression in iPSCs than factors such as somatic tissue of origin. However, the number of individuals in our data set was small, a standard expression quantitative trait loci (eQTL) mapping experiment (see *e.g.*, [15]) was not possible. Therefore we performed the allele-specific expression analysis [2] to identify genes whose expression levels are affected by individual genetic variations and showed that similar effects could be observed in the iPSCs that were previously observed in adult human tissues [15].

For this analysis, we genotyped all our individuals (S2, S4, S5 and S7) except for H9 human ES sample using Illumina HumanOmni2.5-8 BeadChip, followed by imputation with the 1000 Genomes Project data and haplotype phasing using Beagle software [5]. For each individual, we identified all heterozygous exonic SNPs and counted the numbers of fragments separately for each of the two different alleles at the SNP loci in our RNA-seq samples.

Let m_{ijk} and n_{ijk} be the allele-specific fragment count for the mutant allele and the total fragment count at SNP k ($k = 1, \ldots, L_j$) in an exonic region of gene j for the iPSC sample i ($i = 1, \ldots, M$), where i stands for the iPSC RNA-seq sample identifier for an individual (not the identifier for the four individuals in our study). For each gene j, we explicitly modelled the allelic imbalance π_{jk} at heterozygous SNP k using a beta-binomial distribution with an over dispersion parameter θ_j across all iPSC samples from one individual, such that

$$\begin{aligned} m_{ijk} &\sim \mathcal{BB}(n_{ijk}; \pi_{jk}, \theta_j), \\ \text{logit}\, \pi_{jk} &= \beta_j h_{jk}, \end{aligned}$$

where β_j is the common effect size of allelic imbalance for gene j across all SNPs and

$$h_{jk} = \begin{cases} 1 & \text{mutant allele on maternal haplotype,} \\ -1 & \text{mutant allele on paternal haplotype.} \end{cases} \tag{6}$$

Note that maternal/paternal haplotype was arbitrarily determined during the phasing. Then we asked whether the allelic imbalance is significant at gene j in terms of the likelihood ratio P-value between the following two hypotheses:

$$\begin{aligned} H_0 &: \beta_j = 0, \\ H_1 &: \beta_j \neq 0. \end{aligned}$$

The parameter estimation followed by hypothesis testing was performed for each gene, independently. The over dispersion parameter θ_j was estimated by using the modified profile likelihood [4] given the maximum likelihood estimator of β_j, rather than using the maximum likelihood estimator. Because of the small sample size, the maximum likelihood estimator would tend to underestimate the over-dispersion, thereby we would overestimate the number of significant genes with a same FDR threshold.

1.5.1 Comparison with eQTL analysis in Lappalainen *et al.*

For subsequent allele-specific expression analysis, we re-analyzed the RNA-seq data in [15] to identify genes with eQTLs in lymphoblastoid cell lines. We downloaded the raw fastq files for 162 CEU and GBR HapMap individuals at `http://www.geuvadis.org/web/geuvadis`. Reads were aligned and FPKMs were calculated with GC correction as described previously. We performed principal component (PC) analysis to detect unknown confounding factors in the experiment. Then, for each gene, we performed a linear regression of the FPKM values on the first 8 PCs, and replaced the FPKM values with their residuals in that regression as described in [2]. We finally performed cis-eQTL mapping for each gene using a single linear regression with a SNP genotype located within 200Kb from both ends of the gene. The SNP with the minimum P-value for each gene was selected as the cis-eQTL SNP (referred to as eSNP in this text). The high-expression ($+$) and low-expression ($-$) alleles at the eSNP were defined by the sign of regression coefficient. To estimate FDR, we performed permutation of phenotypes as described in [2] and defined eQTL genes and eSNPs by setting study-wide false-discovery rate (FDR) to 5%.

In Figure 2b in the main text, we asked SNP genotypes for our individual at the eSNP and classified those into homozygote of high-expression alleles ($+/+$), heterozygote ($+/-$) and homozygote of low-expression alleles ($-/-$). Then, for genes with an eQTL at an FDR of 5%, we plotted the normalised FPKM $\tilde{y}_{ij}$ against the classified genotypes.

In Figure 2c in the main text, we identified all individuals heterozygous for the eSNPs ($+/-$). Then, in these individuals we classified the "maternal" haplotype into the high-expression haplotype ($+$) or low-expression haplotype ($-$) according to the high/low-expression allele on the haplotype. Finally, for genes with an eQTL at an FDR of 5%, we plotted $\mathrm{logit}^{-1}\hat{\beta}_j$ against the high/low-expression haplotype.

References

[1] Price, A *et al* Principal components analysis corrects for stratification in genome-wide association studies Nature Genetics (2006) 38:904-909

[2] Pickrell, JK *et al*. Understanding mechanisms underlying human gene expression variation with RNA sequencing. Nature (2010) 464:768-772.

[3] `http://www.geneimprint.com/`.

[4] Pawitan Y *In All Likelihood*.

[5] `http://faculty.washington.edu/browning/beagle/beagle.html`.

[6] `http://bowtie-bio.sourceforge.net/bowtie2/index.shtml`.

[7] `http://tophat.cbcb.umd.edu`.

[8] Anders & Huber (2010): Differential expression analysis for sequence count data. Genome Biology 11:R106

[9] Veyrieras *et al*. High-Resolution Mapping of Expression-QTLs Yields Insight into Human Gene Regulation (2008) PLOS Genetics (2008) 4(10):e1000214

[10] Dempster, Laird, Rubin Maximum Likelihood from Incomplete Data via the EM Algorithm Journal of the Royal Statistical Society, Series B (1977) 39:1-38

[11] Geti, I *et al*. A practical and efficient cellular substrate for the generation of induced pluripotent stem cells from adults: blood-derived endothelial progenitor cells. Stem Cells Transl Med. 12:855-865

[12] Alexa & Rahnenfuhrer topGO: topGO: Enrichment analysis for Gene Ontology

[13] Vallier, L *et al* Signaling pathways controlling pluripotency and early cell fate decisions of human induced pluripotent stem cells. Stem Cells (2009) 27:2655-66

[14] Astle, W & Balding DJ Population Structure and Cryptic Relatedness in Genetic Association Studies (2009) Statistical Science 24:451-471

[15] Lappalainen, T *et al* Transcriptome and genome sequencing uncovers functional variation in humans (2013) Nature 501:506511
